# Supplementary material for: Unique universal scaling in nanoindentation pop-ins
Source: Nat Commun. 2020 Aug 21;11:4177. doi: 10.1038/s41467-020-17918-7 (PMC7443148; doi:10.1038/s41467-020-17918-7)
Supplement: Supplementary file 1 — Supplementary Information [file 41467_2020_17918_MOESM1_ESM.pdf]

Supplementary Information

Unique universal scaling in nanoindentation pop-ins

Yuji Sato[1], Shuhei Shinzato[1], Takahito Ohmura[2,3,4,*], Takahiro Hatano[5,**], and Shigenobu Ogata[1,3,***]

[1] Department of Mechanical Science and Bioengineering, Graduate School of Engineering Science, Osaka University, 1-3 Machikaneyama, Toyonaka, Osaka 560-8531, Japan,
[2] Research Center for Structural Materials, National Institute for Materials Science (NIMS), 1-2-1 Sengen, Tsukuba, Ibaraki 305-0047, Japan,
[3] Center for Elements Strategy Initiative for Structural Materials (ESISM), Kyoto University, Yoshida Honmachi, Sakyo, Kyoto 606-8501, Japan,
[4] Graduate School of Engineering, Kyushu University, 744 Motooka, Nishi-ku, Fukuoka 819-0395, Japan,
[5] Department of Earth and Space Science, Graduate School of Science, Osaka University, 1-1 Machikaneyama, Toyonaka, Osaka 560-0043, Japan

[*] OHMURA.Takahito@nims.go.jp
[**] hatano@ess.sci.osaka-u.ac.jp
[***] ogata@me.es.osaka-u.ac.jp

Supplementary Note 1. Verification of the threshold value of displacement burst Δh^{c}

Concerning the discrimination between the pop-in event and electrical and mechanical noise, to investigate displacement difference due to the noise, which mainly consists of many kinds of types including pink noise with $1/f$ distribution, white one with a flat distribution and Gaussian, a noise floor in the captured data with our facilities should be analyzed in probability function. To separate the noise floor from the essential displacement by plastic deformation, a contiguous displacement data should be recorded in a constant applied load with the same capture rate. Since a mechanical noise might depend on the applied load, different peak load conditions should be set. We conducted additional nanoindentation experiments on the (100) surface of BCC Fe with holding peak indentation load at three patterns (10, 100, and 1000 μN) for 100 s, and recorded the displacement as shown in Supplementary Figure 1. The loading rate of the indenter was 50 μNs^{-1}, which is the same as the rate in the nanoindentation experiments in the manuscript, and the sampling rate is 200 points per second. Supplementary Figure 2 shows the magnified curve in the nanoindentation experiment with holding the peak load at 10 μN. There is an irregular variation even under the constant load condition, and this is due to the nanoindentation device by thermal drift. For each case of the peak loads, the displacement differences between the adjacent points during the holding period of 100 s in Supplementary Figure 1 is plotted and shown in Supplementary Figure 3. The number of the captured data is about 20,000 for each condition. Almost all the displacement differences are less than ± 0.4 nm. Since the threshold value in the manuscript was 0.5 nm, we consider that the displacement differences due to only the noise are not included in the results in the manuscript. However, it is possible that the detected displacement burst includes the noise part. Unfortunately, even if we remove the frequency of the noise by conducting frequency analysis based on discrete Fourier transformation, the pop-ins and the noise cannot be separated because plastic deformation events are likely to occur within shorter time than the resolution of the analysis. According to the probability distribution of the noise size as shown in Supplementary Figure 4, the obtained noise is Gaussian noise in any peak load conditions, which is totally different from a pink noise. It should be noted that a white noise is an ideal one as a

kind and/or part of Gaussian noise. Therefore, the noise floor in our system hardly includes a pink noise, and the indentation pop-in data in the manuscript mainly correspond to the mechanical reaction to the applied stress.

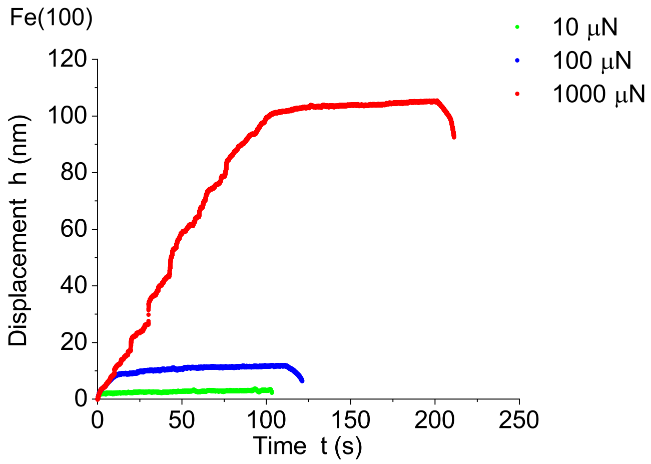

Supplementary Figure 1. Displacement vs. time curves in nanoindentation experiments on the (100) surface of BCC Fe with holding indentation load at three patterns (10, 100, and 1000 μN). The constant loading rate of 50 μNs^{-1} and the same holding period of 100 s.

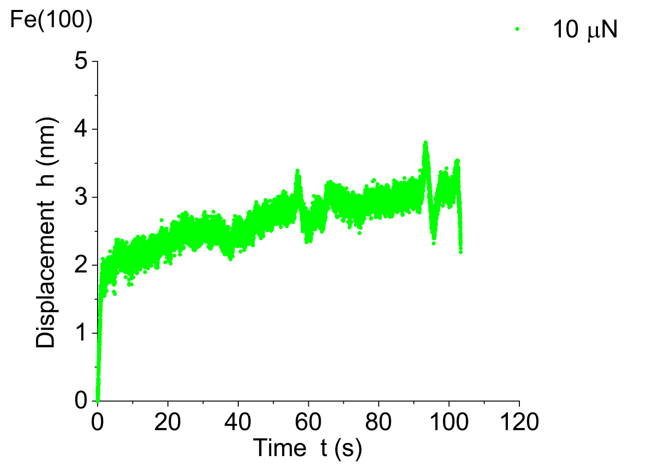

Supplementary Figure 2. Magnified displacement vs. time curves in nanoindentation experiments on the (100) surface of BCC Fe with holding indentation load at 10 μN.

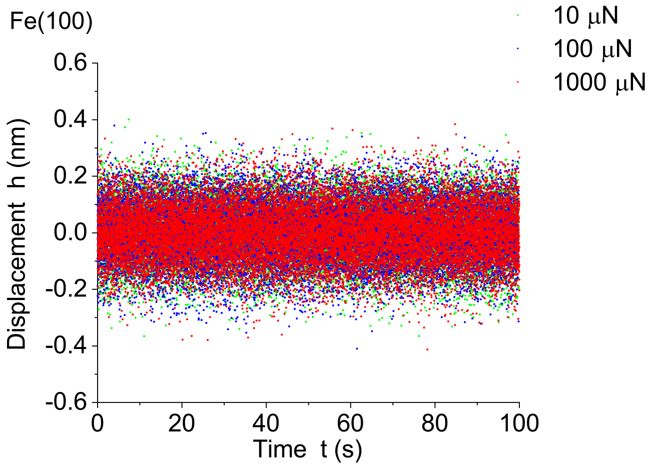

Supplementary Figure 3. Displacement differences in nanoindentation experiments on the (100) surface of BCC Fe with holding indentation load at three patterns (10, 100, and 1000 μN) as a function of hold time.

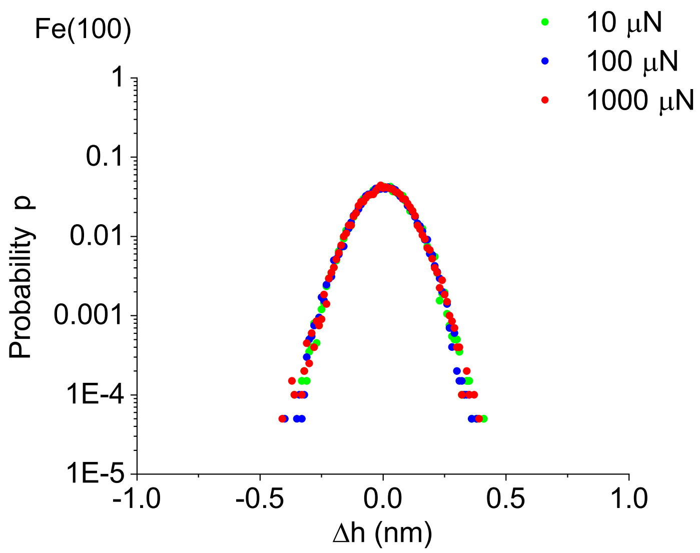

Supplementary Figure 4. Probability distribution of the noise size in the nanoindentation experiments.

Supplementary Note 2. Verification of contact area $A(h)$ within elastic contact region by molecular dynamics simulation

We confirm the Heltz's contact theory [1] using molecular dynamics (MD) simulation. In MD simulation, there is no unique definition and computation method of contact area between contacting two objects because of the discrete nature of MD simulation. Here, we simply but reasonably define the contact area $A(h)$ as $A(h) = N(h) \times \Omega / r_c$, where $r_c = 0.53$ nm is the potential cut-off distance between indenter tip and atoms in the testing materials, Ω is atomic volume in bulk system ($= a^3/n$, $n = 2$ (BCC) and $n = 4$ (FCC), where a is lattice constant), and $N(h)$ is number of atoms in the volume contacting indenter tip, thus atoms within the cut-off distance from the indenter tip. Before first pop-in, $A(h)$ actually follows the Hertz's contact theory πRh [1] as shown in Supplementary Figure 5.

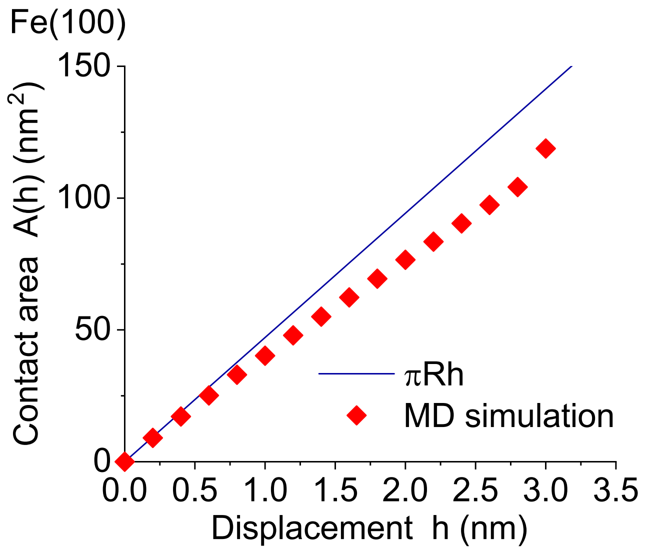

Supplementary Figure 5. Comparison between the contacted area $A(h)$ before first pop-in defined in the manuscript as πRh and $A(h)$ obtained by MD nanoindentation simulation for the (100) surface of BCC Fe.

Supplementary Note 3. Verification of the quadratic area function for estimating contact area $A(h)$

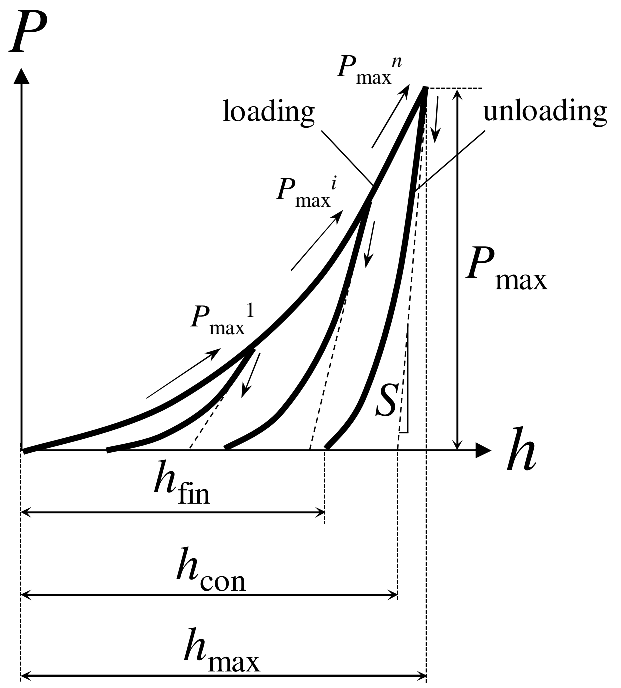

Supplementary Figure 6. Schematic illustration of load P - depth h hysteresis loop curve obtained by nanoindentation for a standard sample like fused silica with isotropic properties. Analysis on unloading curve gives us parameters of the unloading stiffness $S(h)$ and the contact depth $h_{con}(h)$ for $h = h_{max}$ at P_{max}. h_{fin} is the final penetration depth after getting back to zero in load. We vary the P_{max} for $P_{max}{}^{1} \ldots P_{max}{}^{i} \ldots P_{max}{}^{n}$ to get the quadratic area function in Eq. (S4).

The contact area $A(h_{con}(h))$ can be basically calculated by the contact depth $h_{con}(h)$ through the indenter geometry. There are problems for getting accurate values in both parameters. One is the estimation of the $h_{con}(h)$ by separation from elastic displacement in measured depth h, the other is calibration of an imperfection in the indenter geometry. The problems are generally solved through the area function in Oliver-Pharr (OP) method [2], which has been referred in several works for a variety of materials [3, 4, 5, 6, 7]. For the former problem, the con-

tact depth $h_{\mathrm{con}}(h)$ is hard to be calculated because the measured depth h at peak load includes both plastic and elastic displacements. Therefore, the estimation of the $h_{\mathrm{con}}(h)$ by separation from the elastic displacement strongly impinges on an accuracy of the contact area evaluation. The h_{con} can be estimated by an analysis of unloading curve with purely elastic recovery from a peak load as shown schematically in Supplementary Figure 6, which is typically obtained for fused silica as the standard sample. When we have a hysteresis loop of load-depth curve including the segments of loading and subsequent unloading, some parameters are obtained as follows. P_{max} and h_{max} are the measured load and penetration depth at the peak load, respectively. If a contact area between the indenter and sample at P_{max} is kept constant during unloading, a flat-end punch model is applicable to get a load depth relation and then the unloading segment should be rectilinear. The linear line can be drawn with the slope $S = S(h_{\mathrm{max}})$ that is the unloading stiffness analytically given as a differential coefficient at the peak load of a power function fitted with the unloading curve. By extrapolating the linear line to the horizontal axis, the cross point is obtained as the contact depth h_{con}, which corresponds to the penetration depth within the contact region between the indenter and sample at peak load, which is analytically calculated as

$$h_{\mathrm{con}} = h_{\mathrm{max}} - \frac{P_{\mathrm{max}}}{S(h_{\mathrm{max}})}. \tag{S1}$$

Note that the really measured unloading curve generally shows a curved line as shown in Supplementary Figure 6, which means a gradual decreasing in the contact area due to an elastic recovery inside the contact region during unloading, leading to the final penetration depth h_{fin} after getting back to zero in load. Therefore, the h_{fin} is smaller than h_{con} and gives us a wrong value in the contact area at the peak load, and h_{con} should be used instead. For the later problem, we can calibrate the indenter geometry by using the standard sample of fused silica. In the case of an ideal shape of Berkovich indenter in the three-sided pyramid with apex angle of 115°, the contact area $A(h_{\mathrm{con}}(h))$ is given as $A(h_{\mathrm{con}}(h)) = c(h_{\mathrm{con}}(h))^2$ ($c = 24.5$). However, a real tip shape is generally not perfect but truncated by an ablation, and hence the shape should be calibrated. In the OP model, the $S(h)$ is

given as a function of $A(h_{\mathrm{con}}(h))$,

$$S(h) = \frac{2}{\sqrt{\pi}} E_{\mathrm{r}} \sqrt{A(h_{\mathrm{con}}(h))}, \tag{S2}$$

where E_{r} is a reduced elastic modulus and can be written for isotropic material as

$$\frac{1}{E_{\mathrm{r}}} = \frac{1-\nu_{\mathrm{i}}^2}{E_{\mathrm{i}}} - \frac{1-\nu_{\mathrm{s}}^2}{E_{\mathrm{s}}}, \tag{S3}$$

where E and ν are Young's modulus and Poisson's ratio, and subscripts i and s refer to indenter and sample, respectively. For fused silica (isotropic material), E_{r} is 70 GPa [2]. Therefore, the $A(h_{\mathrm{con}}(h_{\mathrm{max}}))$ is calculated by the measurement of $S(h_{\mathrm{max}})$ through Eq. (S1) for the P_{max}. When we vary the P_{max} for ${P_{\mathrm{max}}}^1 \ldots {P_{\mathrm{max}}}^i \ldots {P_{\mathrm{max}}}^n$ as shown in Supplementary Figure 6, we can obtain the corresponding $A(h_{\mathrm{con}}({h_{\mathrm{max}}}^1)) \ldots A(h_{\mathrm{con}}({h_{\mathrm{max}}}^i)) \ldots A(h_{\mathrm{con}}({h_{\mathrm{max}}}^n))$. Also, the corresponding $h_{\mathrm{con}}({h_{\mathrm{max}}}^1) \ldots h_{\mathrm{con}}({h_{\mathrm{max}}}^i) \ldots h_{\mathrm{con}}({h_{\mathrm{max}}}^n)$ can be obtained through Eq. (S1). By plotting the $A(h_{\mathrm{con}}(h))$ vs. $h_{\mathrm{con}}(h)$, we can obtain the area function by fitting to the standard quadratic function given in

$$A(h) = A(h_{\mathrm{con}}(h)) = c_2(h_{\mathrm{con}}(h))^2 + c_1 h_{\mathrm{con}}(h) + c_{\frac{1}{2}}(h_{\mathrm{con}}(h))^{\frac{1}{2}}, \tag{S4}$$

where $c_2 = 24.5$, $c_1 = 2.61 \times 10^3$ nm, and $c_{\frac{1}{2}} = 1.57 \times 10^{-7}$ $\mathrm{nm}^{\frac{3}{2}}$.

The second and third terms in the quadratic area function are the correction term for the imperfect tip. Once we get the area function of the indenter geometry, it can be used for every material universally, and E_{r} can be evaluate through a measurement of $S(h)$. For making sure that the quadratic area function works for the Fe single crystal, we conducted additional nanoindentation experiments with setting four different peak loads (250, 500, 750, and 1000 μN) on the (100) surface of the BCC Fe. We measured $A'(h_{\mathrm{fin}}(h))$ as a contact area by a direct measurement on the atomic force microscope (AFM) image instead of $S(h)$ measurement for cross checking, and then $A'(h_{\mathrm{fin}}(h))$ is compared with $A(h_{\mathrm{con}}(h))$ that is independently given by $h_{\mathrm{con}}(h)$ through the quadratic area function in Eq. (S4). Note that the area function $A'(h_{\mathrm{fin}}(h))$ is different from $A(h_{\mathrm{con}}(h))$ because the geometry of the indent print is different due to the elastic recovery within the contact region, but both functions follow the quadratic function basically. The AFM

images of the surface imprint in each nanoindentation are shown in the following Supplementary Figure 7. The image is presented in a gradient mode with 256×256 pixels^2, in which the contrast corresponds to a local cant on the surface. The triangle imprints are clearly shown and the three vertexes or sides of the triangle are determined. The triangles size increases as the peak load increases. However, the top view image cannot give us a 3D information for the contact area. Therefore, the cross-section profiles of the imprints were measured on the topographic image as shown in Supplementary Figure 8. The maximum depth is given in each case, which corresponds to the $h_{\text{fin}}(h)$ in Supplementary Figure 6. Additionally, the distance between the red and green dots on the topographic image can be measured as a representative horizontal size of the imprint. By using the values of horizontal and vertical sizes, the contact area $A^{'}(h_{\text{fin}}(h)) = c \times (h_{\text{fin}}(h))^2$ can be calculated based on an assumption of regular triangular pyramid for the four load conditions. The plot of the independently calculated $A^{'}(h_{\text{fin}}(h))$ and $A(h_{\text{con}}(h))$ are shown in Supplementary Figure 9. The plots fit well with a linear line, meaning the area function in the quadratic function works in Fe. It should be noted that the $A^{'}(h_{\text{fin}}(h))$ is lower about 5% than $A(h_{\text{con}}(h))$ with the same P_{max} condition because the $A(h_{\text{con}}(h))$ includes an elastic displacement under the maximum load while $A^{'}(h_{\text{fin}}(h))$ is in the condition after unloading without any elastic deformation. When we calculate $\Delta\sigma$ at the end of the first pop-in, at the beginning and at the end of the second and subsequent pop-in, we used $A(h_{\text{con}}(h))$ in Eq. (S1) for a strict evaluation. To get the $A(h_{\text{con}}(h))$ though Eq. (S1), the $S(h)$ is necessary by unloading analysis. Since the $S(h)$ cannot be measured in the case on a continuous loading segment without unloading, the $A(h_{\text{con}}(h))$ is numerically solved on the combination of Eqs. (S1) and (S2) by using only P as P_{max} and h as h_{max} on the loading segment. Note that the E_{r} for Fe and Cu samples in Eq. (S2) is evaluated in each sample as given in Methods section.

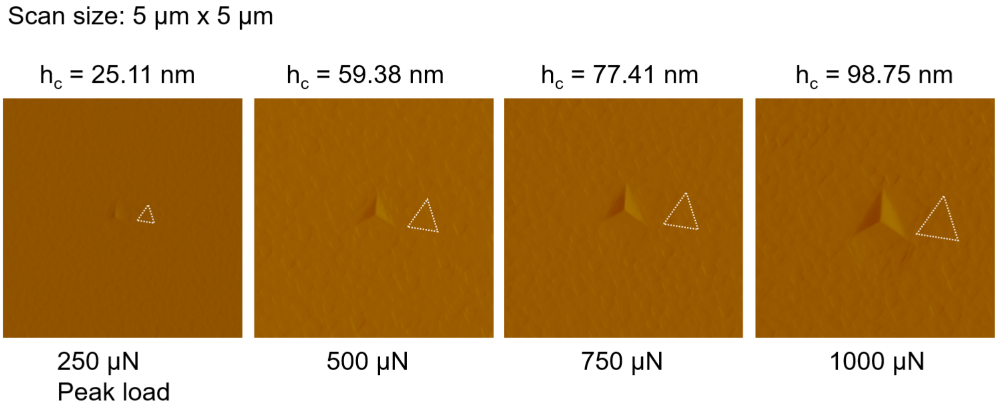

Supplementary Figure 7. Atomic force microscope (AFM) images of the (100) surface of the BCC Fe sample with an indent. The scan area was $5 \times 5\ \mu m^2$. The broken lines are the approximation of surface imprint by equilateral triangle.

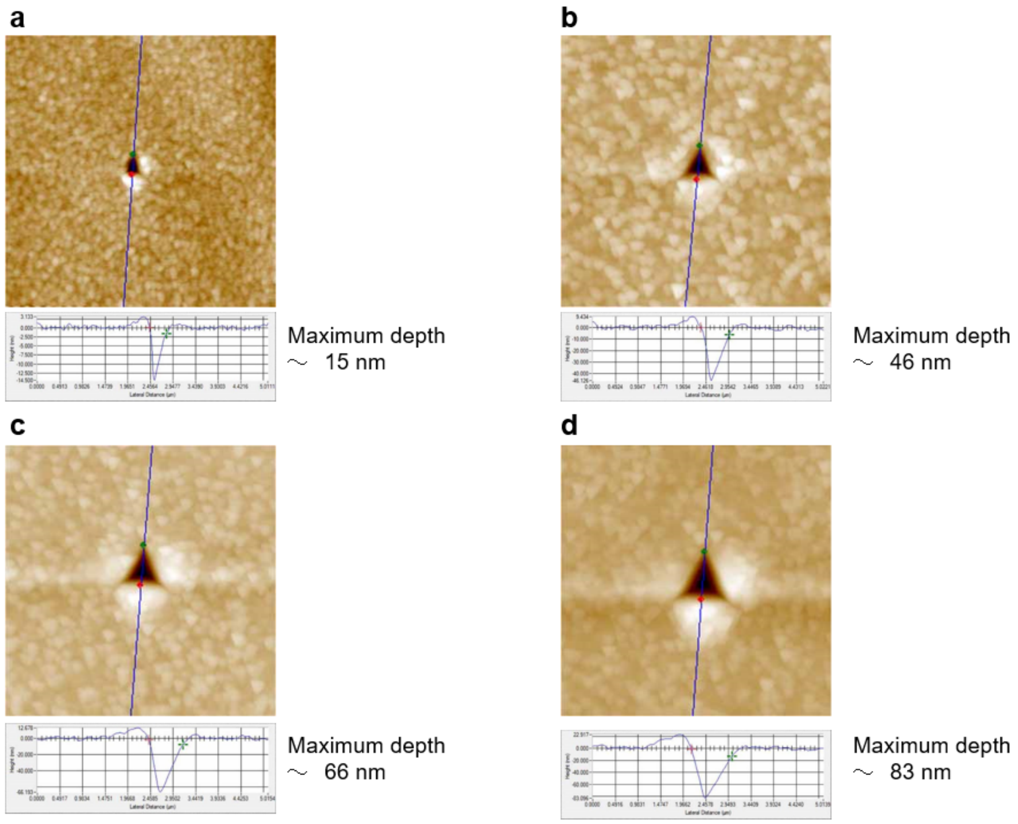

Supplementary Figure 8. AFM topographic images and cross section profiles of the indent imprints of (a) 250 μN, (b) 500 μN, (c) 750 μN, and (d) 1000 μN load conditions. The origin in the vertical axis of the cross-section profile is the original height of the sample surface. We can determine the edge of the triangle as the red and green cross positions. The distance between them can be measured as a representative horizontal size. Also, the maximum depth of the imprint is given in the profile. By using the values of horizontal and vertical sizes, the contact area $A'(h_{\text{fin}})$ can be calculated based on an assumption of regular triangular pyramid.

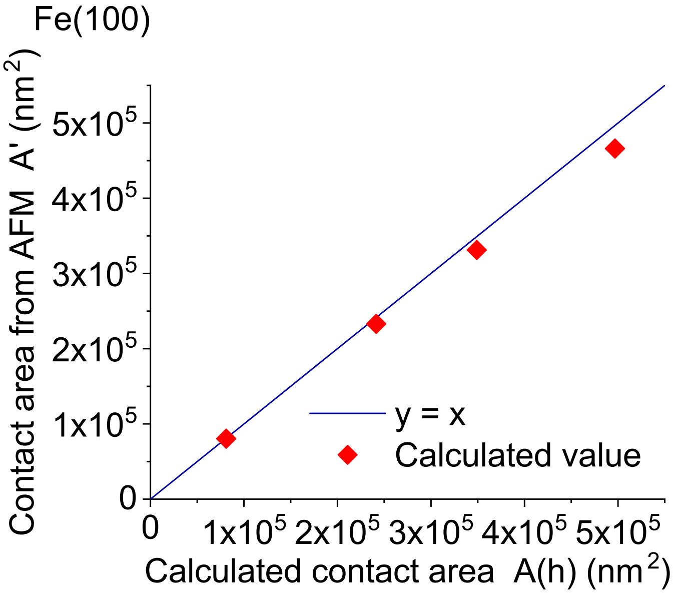

Supplementary Figure 9. The calculated contact area $A(h)$ from the contact depth after unloading h in load-displacement curve vs. the contact area A' from the AFM images.

Supplementary Note 4. Probability distributions of first and subsequent pop-in magnitudes as function of the stress drop $\Delta\sigma$

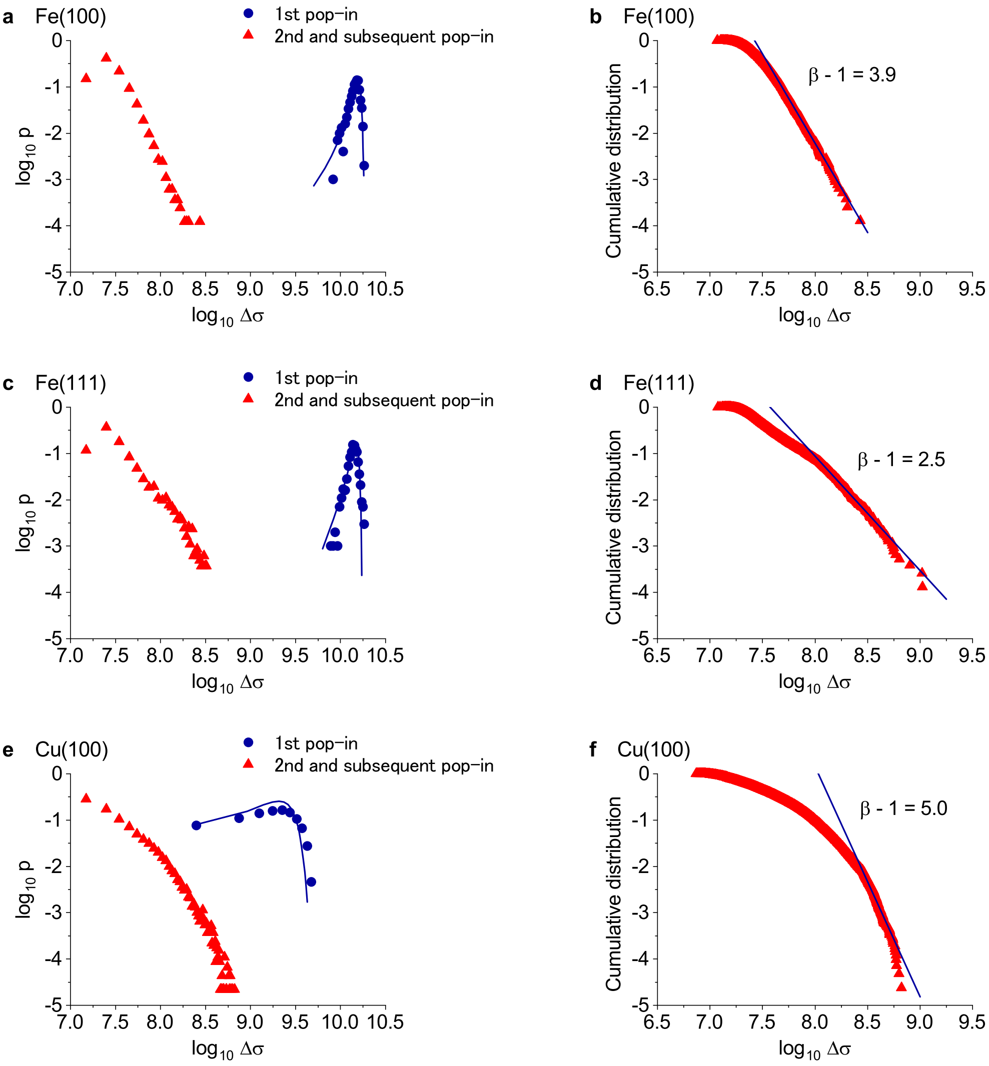

Supplementary Figure 10. Probability distributions of pop-in magnitudes as a function of the stress drop. (a)(c)(e) Probability distributions of first pop-in magnitudes as a function of the stress drop $\Delta\sigma$ (Pa) for the (a) (100), (c) (111) surfaces of BCC Fe, and (e) the (100) surface of FCC Cu, obtained by equal-width binning (5.0×10^8 Pa for first pop-in and 1.0×10^7 Pa for second and subsequent pop-ins). The solid lines represent the fitting curves based on thermal activation theory, Eq. (2) (see text). For reference, (a)(c)(e) are shown, which are the same as those of Figure 2 in the main text. (b)(d)(f) Probability distributions of subsequent pop-in magnitudes as a function of the stress drop $\Delta\sigma$ (Pa) for the (b) (100), (d) (111) surfaces of BCC Fe, and (f) the (100) surface of FCC Cu, obtained by bin-free cumulative distribution. The power law exponents were estimated by a least square fitting using the data within $7.7 \leq \log_{10}\Delta\sigma$ for BCC Fe (100), $8.0 \leq \log_{10}\Delta\sigma$ for BCC Fe (111), and $8.4 \leq \log_{10}\Delta\sigma$ for FCC Cu (100).

Supplementary Note 5. Bin-free cumulative, equal-width binning, and logarithmic binning distributions of subsequent pop-in magnitude

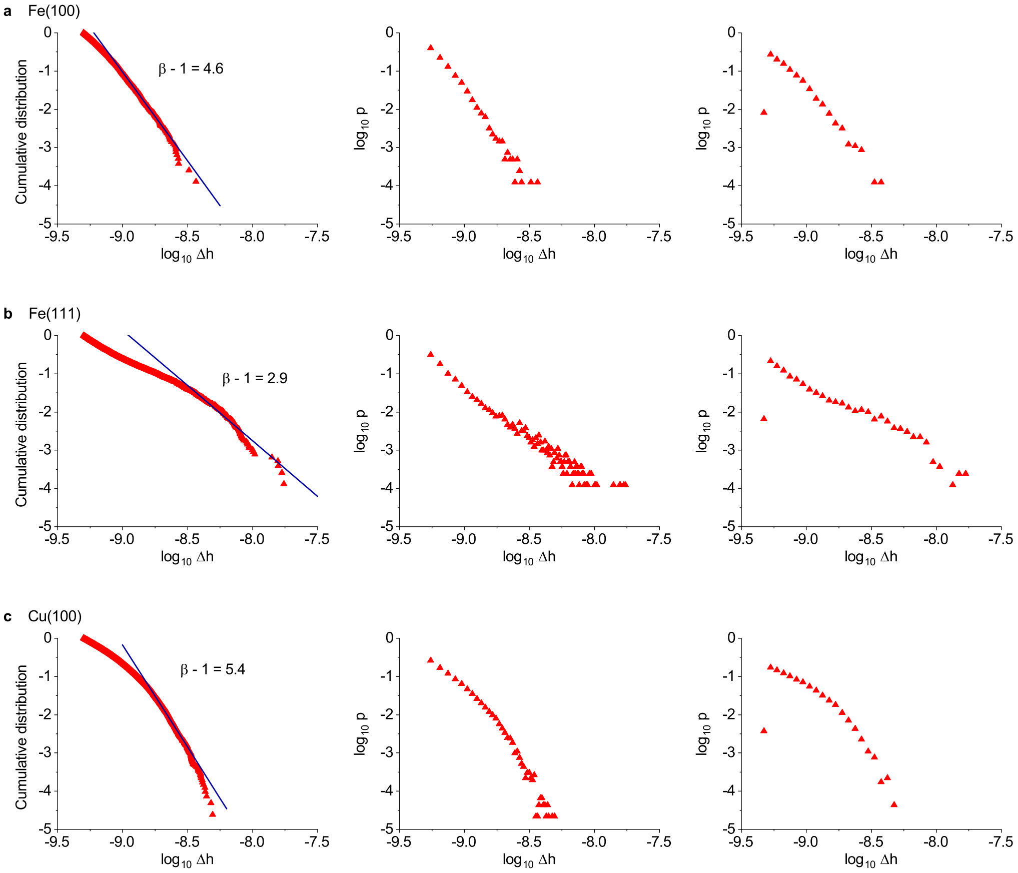

Supplementary Figure 11. Probability distributions of subsequent pop-in magnitudes. Bin-free cumulative (left panels), equal-width binning (middle panels), and logarithmic binning (right panels) distributions of subsequent pop-in magnitude as a function of the displacement burst Δh (m) for the (a) $\langle 100 \rangle$ and (b) $\langle 111 \rangle$ surfaces of BCC Fe and (c) the $\langle 100 \rangle$ surface of FCC Cu. For reference, the bin-free cumulative distributions (left panels) are shown, which are the same as those of Figure 2 in the main text.

Supplementary Note 6. Prediction model of first pop-in probability distribution

In our recent study, we suggested the prediction model of first pop-in probability distribution [8]. Diverting the prediction model, we conducted fitting of the experimental first pop-in distribution for the (100) and (111) surfaces of BCC Fe and the (100) surface of FCC Cu.

A residence probability $S(t)$ that the first pop-in does not take place in whole system during time t is given by [9]:

$$\frac{\mathrm{d}S(t)}{\mathrm{d}t} = -k(t)S(t), \tag{S5}$$

$$S(t) = \frac{1}{C}\exp\left[-\int_0^t k(t')\mathrm{d}t'\right]. \tag{S6}$$

Based on transition state theory,

$$k(t) = \sum_i^N k_0(\boldsymbol{R}_i)\exp\left(-\frac{G(t,\boldsymbol{R}_i)}{k_\mathrm{B}T}\right), \tag{S7}$$

where, k_0 is attempt frequency, k_B is the Boltzmann constant, T is absolute temperature, $G(t,\boldsymbol{R}_i)$ is the activation free energy, $\boldsymbol{R}_i$ is position of possible dislocation nucleation site i, and N is number of the possible nucleation sites. The pop-in probability distribution $p(t)$ is given by,

$$p(t) = -\frac{\mathrm{d}S(t)}{\mathrm{d}t} = \frac{1}{C}k(t)\exp\left[-\int_0^t k(t')\mathrm{d}t'\right]. \tag{S8}$$

Because of

$$\int_0^{t_\mathrm{c}} p(t)\mathrm{d}t = 1, \tag{S9}$$

$$C = \int_0^{t_\mathrm{c}} k(t)\exp\left[-\int_0^t k(t')\mathrm{d}t'\right]\mathrm{d}t, \tag{S10}$$

where, t_c is the maximum residence time at a given loading rate $\dot{P}(t)$. $p(t)$ can be

rewritten as a function of the load $P(t)$,

$$p(P^{\text{pop-in}}) = \frac{k(P^{\text{pop-in}})\exp\left[-K\int_0^{P^{\text{pop-in}}} k(P')\mathrm{d}P'\right]}{\int_0^{P_c^{\text{pop-in}}} k(P^{\text{pop-in}})\exp\left[-K\int_0^{P^{\text{pop-in}}} k(P')\mathrm{d}P'\right]\mathrm{d}P^{\text{pop-in}}}, \quad \text{(S11)}$$

where $K = \dot{P}^{-1}$ for the constant loading rate $\dot{P}$ and $K = 1/(2\dot{h}E_r^{1/2})(3P/4E_rR^{1/2})^{-1/3}$ for the constant displacement rate (velocity) $\dot{h}$ using Heltz's contact theory [1].

To address the effect of pre-existing immobile defects on the first pop-in probability, we considered the dislocation nucleation rate from immobile defects $k^{\mathrm{D}}(P^{\text{pop-in}})$, in addition to that from homogeneous nucleation sites $k^{\mathrm{H}}(P^{\text{pop-in}})$. Thus,

$$k(P^{\text{pop-in}}) = k^{\mathrm{H}}(P^{\text{pop-in}}) + k^{\mathrm{D}}(P^{\text{pop-in}}), \quad \text{(S12)}$$

where

$$k^{\mathrm{H}}(P^{\text{pop-in}}) = \sum_i^{N-N^{\mathrm{D}}} k_0(\boldsymbol{R}_i)\exp\left(-\frac{G(P^{\text{pop-in}},\boldsymbol{R}_i)}{k_{\mathrm{B}}T}\right), \quad \text{(S13)}$$

$$k^{\mathrm{D}}(P^{\text{pop-in}}) = \sum_i^{N^{\mathrm{D}}} k_0^{\mathrm{D}}(\boldsymbol{R}_i)\exp\left(-\frac{G_i^{\mathrm{D}}(P^{\text{pop-in}},\boldsymbol{R}_i)}{k_{\mathrm{B}}T}\right), \quad \text{(S14)}$$

$$G(P^{\text{pop-in}},\boldsymbol{R}_i) = E(P^{\text{pop-in}},\boldsymbol{R}_i)\left(1-\frac{T}{T_{\mathrm{m}}}\right). \quad \text{(S15)}$$

while $G(P^{\text{pop-in}},\boldsymbol{R}_i)$ is the activation free energy with a temperature-dependent factor $(1-T/T_{\mathrm{m}})$ [10], where T_{m} is the melting temperature, $G_i^{\mathrm{D}}(P^{\text{pop-in}},\boldsymbol{R}_i)$ is the activation free energy of dislocation nucleation from the defect i, $k_0(\boldsymbol{R}_i)$ and $k_0^{\mathrm{D}}(\boldsymbol{R}_i)$ are the attempt frequencies at homogeneous and heterogeneous nucleation sites i, respectively, and N^{D} is the number of possible heterogeneous nucleation sites in the target material, which should be on the order of the number of atoms in the model. $E(P^{\text{pop-in}},\boldsymbol{R}_i)$ is the activation energy at 0 K at nucleation site i. We assumed that the activation barrier is uniform at the all nucleation site, and $E(P^{\text{pop-in}})$ can be written in the form of Kocks and co-workers [11]; $E(P^{\text{pop-in}}) = E_0\{1-(P^{\text{pop-in}}/P_c^{\text{pop-in}})^u\}^v$, where E_0 is the activation energy under the stress-free condition, and u and v are exponential parameters. In the recent study, we assumed that the easiest nucleation sites (the maximum-resolved-shear-

stress-sites) dominate the nucleation rate $k^{\mathrm{H}}(P^{\text{pop-in}})$ because the high-shear-stress spot is spatially well localized and used the fact that the dislocation nucleation rate is rapidly decreasing with decreasing the shear stress [12]; thus,

$$k^{\mathrm{H}}(P^{\text{pop-in}}) \approx N_{\mathrm{eq}} k_0(\boldsymbol{R}_{\mathrm{MRSS}}(P^{\text{pop-in}})) \exp\left(-\frac{G(P^{\text{pop-in}}, \boldsymbol{R}_{\mathrm{MRSS}}(P^{\text{pop-in}}))}{k_{\mathrm{B}}T}\right), \tag{S16}$$

where, in our nanoindentation configuration, $N_{\mathrm{eq}} = 8$, which is equal to the number of equivalent slip systems, $\boldsymbol{R}_{\mathrm{MRSS}}(P^{\text{pop-in}})$ is the position that exhibits the maximum resolved shear stress at an indentation load $P^{\text{pop-in}}$.

Concerning the dislocation nucleation rate from the immobile defects $k^{\mathrm{D}}(P^{\text{pop-in}})$, we assumed the equivalence of all of the pre-existing immobile defects and these defects are randomly distributed in the target material. Under this assumption, an expectation value of dislocation nucleation rate at a defect is written as,

$$\bar{k}^{\mathrm{D}} = \int_{\boldsymbol{\sigma}} \rho(\boldsymbol{\sigma}) \tilde{k}^{\mathrm{D}}(\boldsymbol{\sigma}) \mathrm{d}\boldsymbol{\sigma}, \tag{S17}$$

where the relation $\boldsymbol{\sigma} = \boldsymbol{\sigma}(P^{\text{pop-in}}, \boldsymbol{R})$ is the atomic stress tensor. $\rho(\boldsymbol{\sigma})$ is a stress probability density distribution in the target material;

$$\rho(\boldsymbol{\sigma}) = \frac{1}{V} \int_V \delta(\boldsymbol{\sigma}'(P^{\text{pop-in}}, \boldsymbol{R}) - \boldsymbol{\sigma}) \mathrm{d}\boldsymbol{R}, \tag{S18}$$

where δ is Dirac's delta function. $\tilde{k}^{\mathrm{D}}$ is dislocation nucleation rate at the considering defect. Since the number of heterogeneous nucleation sites N^{D} is the product of the defect number density ρ^{D} and the volume of target material V, Eq. (S14) is rewritten as,

$$k^{\mathrm{D}}(P^{\text{pop-in}}) = \rho^{\mathrm{D}} V \bar{k}^{\mathrm{D}}. \tag{S19}$$

In atomistic modeling, $k^{\mathrm{D}}(P^{\text{pop-in}})$ is computed using the obtained atomic stress distribution and $\rho(\boldsymbol{\sigma})$ is computed in a discrete manner, such as

$$\rho(\boldsymbol{\sigma}) \approx \frac{1}{V} \sum_{i}^{N_{\mathrm{atom}}} \delta(\boldsymbol{\sigma}_i' - \boldsymbol{\sigma}) \Omega_i, \tag{S20}$$

where $\boldsymbol{\sigma}_i^{'}$ and Ω_i are the atomic stress and atomic volume at atom i, respectively. N_{atom} is total number of atoms in the system.

For simplicity, we approximated $\bar{k}^{\mathrm{D}}$ by

$$\bar{k}^{\mathrm{D}}(\boldsymbol{\sigma}) \approx \int_0^{\tau^{\mathrm{MRSS}}} \rho(\tau^{\mathrm{RSS}}) k^{\mathrm{D}}(\tau^{\mathrm{RSS}}) \mathrm{d}\tau^{\mathrm{RSS}}, \tag{S21}$$

$$k^{\mathrm{D}}(\tau^{\mathrm{RSS}}) = k_0^{\mathrm{D}} \exp\left(-\frac{G^{\mathrm{D}}(\tau^{\mathrm{RSS}})}{k_{\mathrm{B}}T}\right), \tag{S22}$$

$$G^{\mathrm{D}}(\tau^{\mathrm{RSS}}) = E^{\mathrm{D}}(\tau^{\mathrm{RSS}}) \left(1 - \frac{T}{T_{\mathrm{m}}^{\mathrm{D}}}\right), \tag{S23}$$

with ignoring the effect of multicomponent stress on activation free energy G^{D}. Where τ^{RSS} is the resolved shear stress component of $\boldsymbol{\sigma}$. $E^{\mathrm{D}}(\tau^{\mathrm{RSS}})$ is the activation energy at 0 K; $E^{\mathrm{D}}(\tau^{\mathrm{RSS}}) = E_0^{\mathrm{D}}\{1 - (\tau^{\mathrm{RSS}}/\tau_{\mathrm{max}}^{\mathrm{CRSS}^{\mathrm{D}}})^{u^{\mathrm{D}}}\}^{v^{\mathrm{D}}}$, where E_0^{D} is the activation energy of heterogeneous dislocation nucleation under the stress-free condition, u^{D} and v^{D} are exponential parameters, and $\tau_{\mathrm{max}}^{\mathrm{CRSS}^{\mathrm{D}}}$ is the critical resolved shear stress of heterogeneous dislocation nucleation on $(1\bar{1}0)[111]$ slip system for BCC Fe and $(1\bar{1}1)[110]$ slip system for FCC Cu. $T_{\mathrm{m}}^{\mathrm{D}}$ is the melting temperature at the considering defect and approximately set to the same value of T_{m}.

In this fitting, k_0 is set to 10^{13} s^{-1} [9], T_{m} is 1811 K for Fe [13] and 1356 K for Cu [14], the parameters in $E(P^{\text{pop-in}})$ are set to the values in our recent study [8] ($E_0 = 31744.2$ eV, $u = 0.0418778$, $v = 3.67947$, $P_{\mathrm{c}}^{\text{pop-in}} = 3.073338$ μN) for Fe and in newly fitted values ($E_0 = 25000.0$ eV, $u = 0.0418778$, $v = 3.67947$, $P_{\mathrm{c}}^{\text{pop-in}} = 0.77615$ μN) for Cu, and the calculation result in our recent study is employed as $\rho(\boldsymbol{\sigma})$. We tuned C, $\sigma_{\mathrm{c}}^{\mathrm{contact}}$, and the parameters involved in heterogeneous dislocation nucleation from immobile defects – k_0^{D}, ρ^{D}, E_0^{D}, $\tau_{\mathrm{max}}^{\mathrm{CRSS}^{\mathrm{D}}}$, u^{D}, and v^{D} –. The fitting parameters are shown in Supplementary Table 1. We used Newton-Raphson method for the fitting.

Supplementary Table 1. Value of fitting parameters for the experimental first pop-in distribution.

Surface orientation	Fe(100)	Fe(111)	Cu(100)
$C\,(\mathrm{Pa/m})^{\frac{2}{3}}$	2.12×10^{11}	2.05×10^{11}	1.10×10^{11}
$\sigma_c^{\mathrm{contact}}$ (GPa)	1.6	1.4	2.3
$k_0^{\mathrm{D}}\,(\mathrm{s}^{-1})$	1.5×10^{13}	1.5×10^{13}	1.5×10^{13}
$\rho^{\mathrm{D}}\,(\mathrm{m}^{-3})$	6.0×10^{24}	6.0×10^{24}	4.0×10^{24}
E_0^{D} (eV)	47.0	47.0	10.0
$\tau_{\max}^{\mathrm{CRSS}^{\mathrm{D}}}$ (GPa)	2.6	2.6	0.6
u^{D}	0.67	0.67	0.80
v^{D}	1.13	1.13	1.35

Supplementary Note 7. First pop-in probability distribution in linear scale

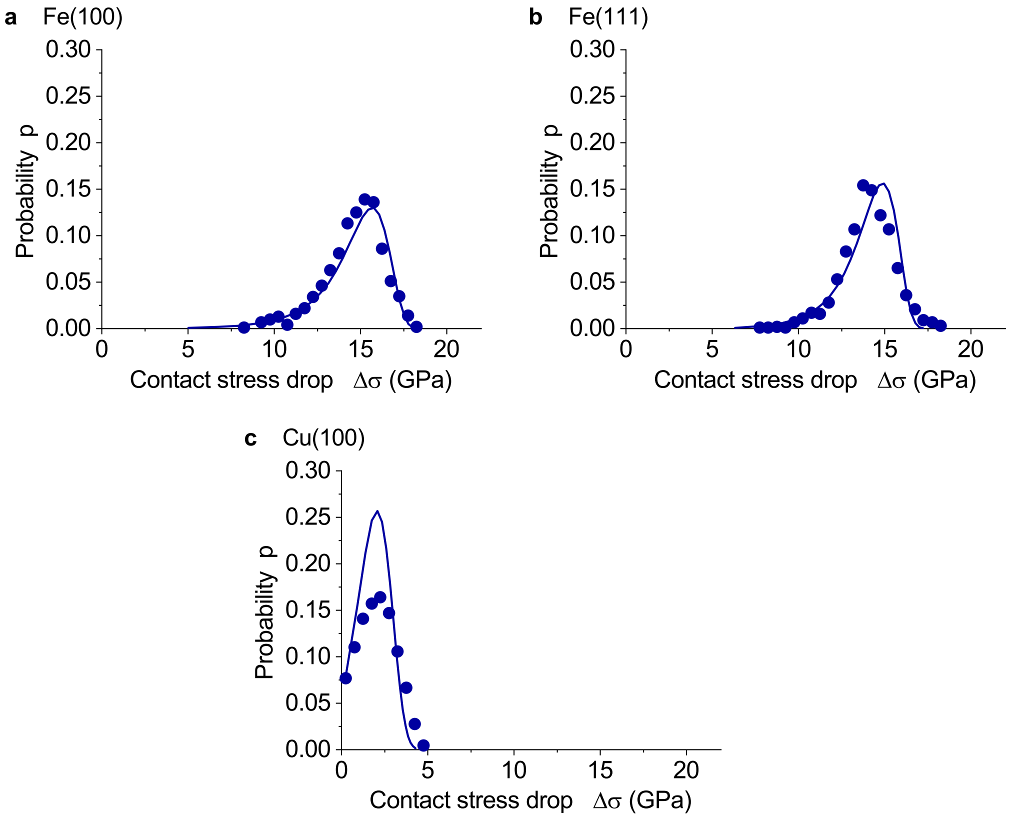

Supplementary Figure 12. Probability distributions of first pop-in magnitude as a function of the contact stress drop $\Delta\sigma$ (Pa) for the (a) (100) and (b) (111) surfaces of BCC Fe and (c) the (100) surface of FCC Cu in linear scale. The solid lines represent the fitting curves based on thermal activation theory, Eq. (2) (see text).

Supplementary Note 8. Temperature and loading rate dependencies of the probability distribution of first pop-in magnitudes

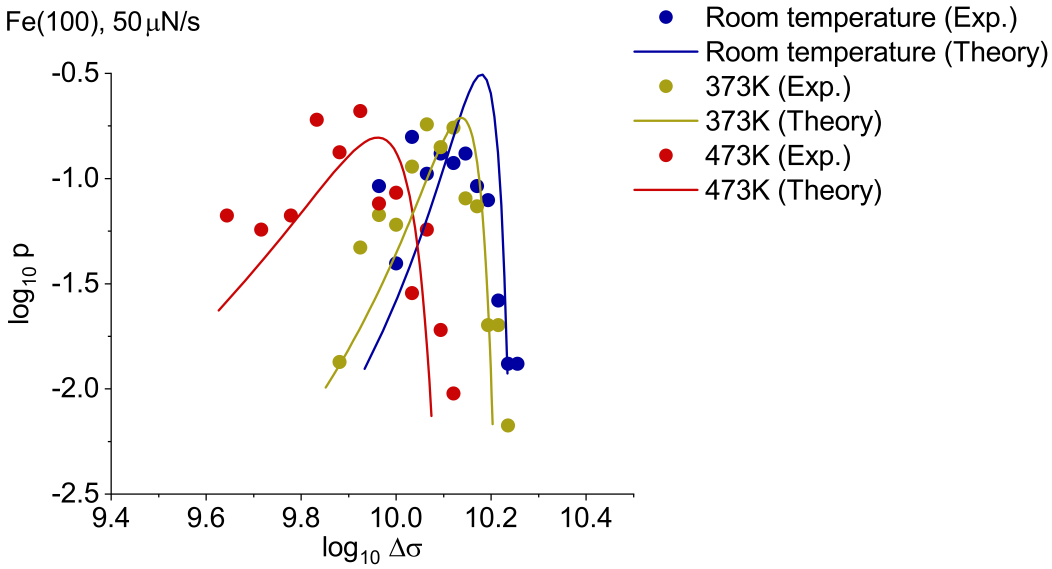

Supplementary Figure 13. Probability distributions of first pop-in magnitude as a function of the contact stress drop $\Delta\sigma$ (Pa) for the (100) surface of BCC Fe obtained from nanoindentation experiments at room temperature, 373 and 473 K (equal-width binning, 8.0×10^{8} Pa) with 50 μNs^{-1}. The solid lines represent the fitting curves based on thermal activation theory, Eq. (2) (see text). The fitting was done for room temperature ($= 300$ K) first, then only the temperature and defect density in the Eq. (2) was changed to draw the other temperature curves. The changed parameters in Supplementary Table 1 are $C = 2.40 \times 10^{11}$ $(\mathrm{Pam}^{-1})^{\frac{2}{3}}$, $\rho^{\mathrm{D}} = 6.0 \times 10^{24}$ (m^{-3}) (300 K), $\rho^{\mathrm{D}} = 6.0 \times 10^{22}$ (m^{-3}) (373 K), and $\rho^{\mathrm{D}} = 1.0 \times 10^{22}$ (m^{-3}) (473 K). The change in the defect density implies that the immobile defect density was decreased with the increase of the temperature because of an annealing. Note that these nanoindentation tests for temperature dependency analysis were performed using different nanoindentation machine from other tests as we describe later. Therefore, even at the same room temperature, the data (only 83 data) were different from the data shown in Figure 2 in the main text and Supplementary Figure 14, and thus the Gaussian-like distribution is also slightly different even at the same temperature.

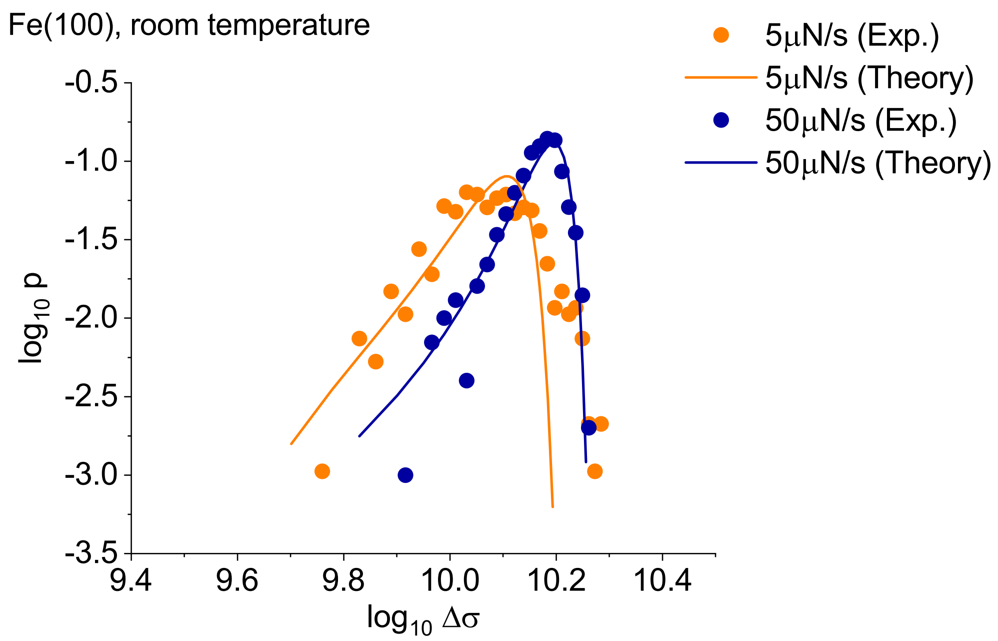

Supplementary Figure 14. Probability distributions of first pop-in magnitude as a function of the contact stress drop $\Delta\sigma$ (Pa) for the (100) surface of BCC Fe obtained from nanoindentation experiments at 5 and 50 μNs^{-1} with room temperature (equal-width binning, 5.0×10^8 Pa). The solid lines represent the fitting curves based on thermal activation theory, Eq. (2) (see text). The fitting was done for 50 μNs^{-1} first (Supplementary Table 1), then only the loading rate in the Eq. (2) was changed to draw the 5 μNs^{-1} curve.

Nanoindentation experiment at elevated temperatures

Above nanoindentation measurements at low loading rate has been done the same testing machine and method as described in Methods section in the manuscript. On the other hand, above nanoindentation measurements at elevated temperatures were done by a high-temperature stage (Bruker Co.) placed in a vacuum chamber on a vibration isolation stage (Minus K Technology Inc.) [15]. Note that this is different from the nanoindentation testing machine we have used to obtain the data in Figure 2 in the main manuscript. The inner environment in the vacuum chamber was controlled by gas introduction through gas inlets. The chamber was

cyclically evacuated up to 1.33 mPa (10^{-5} Torr) and backfilled with argon gas (99.9999 % purity) to reduce the oxygen partial pressure before heating up. A sample was heated from both top and bottom sides to uniform and stabilize the temperature. A diamond indenter was heated passively at 100 μm height from the sample surface together with the sample. The sample and tip were held at the target temperature for 1-2 hours to stabilize the temperature before starting nanoindentation measurements. Additionally, the indenter tip was touched on the sample surface with a small load of about 1 μN for 180 s to ensure the better thermal equilibrium before each nanoindentation measurements. The thermal drift rate was controlled within 0.01 ± 0.03 nms^{-1} in the case at 473 K. The loading and unloading sequence were the same with those at room temperature (RT). Several indentation positions with 20×20 μm^2 in size were selected randomly on the sample surface, and 36 indents were made in each regions with a pitch of 4 μm, which is smaller than the condition in RT to save a space within the smaller heated window but still large enough to avoid interactions between the indent marks with typical horizontal size of 0.7 μm. Some of load-displacement data with unusual behavior that is deviated remarkably from the others were eliminated by visual check in each curve. The measurements at RT with the high-temperature stage were also done to check a coincidence with the data with the RT device. The averages with standard deviation of the measured Young's moduli calculated from unloading segment were 180 ± 18.3, 177 ± 18.7, and 173 ± 21.6 GPa for RT, 373, and 473 K, respectively, which are slightly lower but roughly consistent with literature values and confirm a certain accuracy of the results. The oxidation of the sample surface was significant above 473 K, and measurements could not be done to get a reasonable data at the temperature range.

Supplementary Note 9. Probability distributions of pop-in magnitudes obtained from displacement-controlled MD nanoindentation

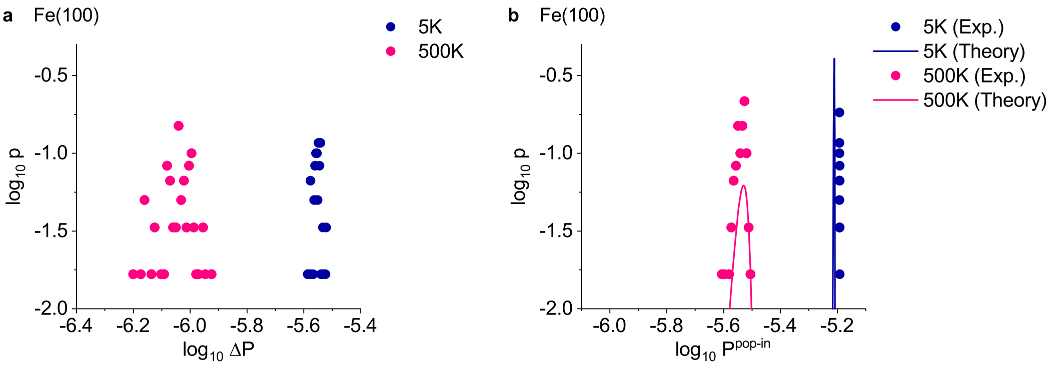

Supplementary Figure 15. Probability distributions of first pop-in magnitude as a function of (a) force drop ΔP (N) and (b) pop-in load $P^{\text{pop-in}}$ (N) for the (100) surface of BCC Fe obtained from 60 MD nanoindentation simulations at 5 and 500 K. The solid lines in the pop-in load plot represent the fitting curves based on the homogeneous dislocation nucleation theory (Eq. (2) (see text) and [8]) using fitted parameters, $u = 0.0035$, $v = 1.85$, and $P_c = 6.514935$ μN. Only these three parameters were changed from the paper [8] owing to the difference of the indenter tip radius.

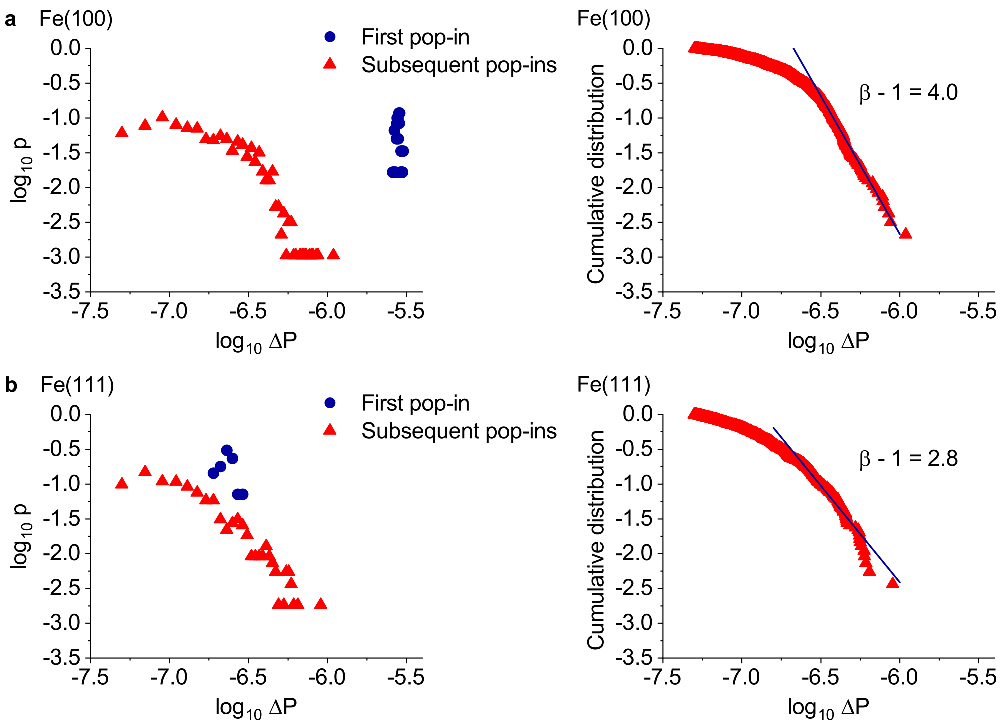

Supplementary Figure 16. Probability distributions of pop-in magnitudes as a function of the indentation force drop ΔP (N) for the Fe (a) (100), (b) (111) surfaces obtained from displacement-controlled 60 MD nanoindentation simulations at 5 K (equal-width binning, 0.02 μN) giving 944 for BCC Fe (100) and 547 for BCC Fe (111) subsequent pop-ins within the statistical independent range (all of the data included other than the first force drop) (See Supplementary Figures 18 and 19). Bin-free cumulative distributions for the subsequent pop-ins are also shown in the right panel. The power law exponent was estimated by least square fitting using the data within -6.5 $\leq \log_{10}\Delta P$ for BCC Fe (100) and -6.7 $\leq \log_{10}\Delta P$ for BCC Fe (111). Note that because of the limited model size in MD simulation, large-scale events should be truncated and thus power-law behavior can be seen within a limited range compared with those of the experiments.

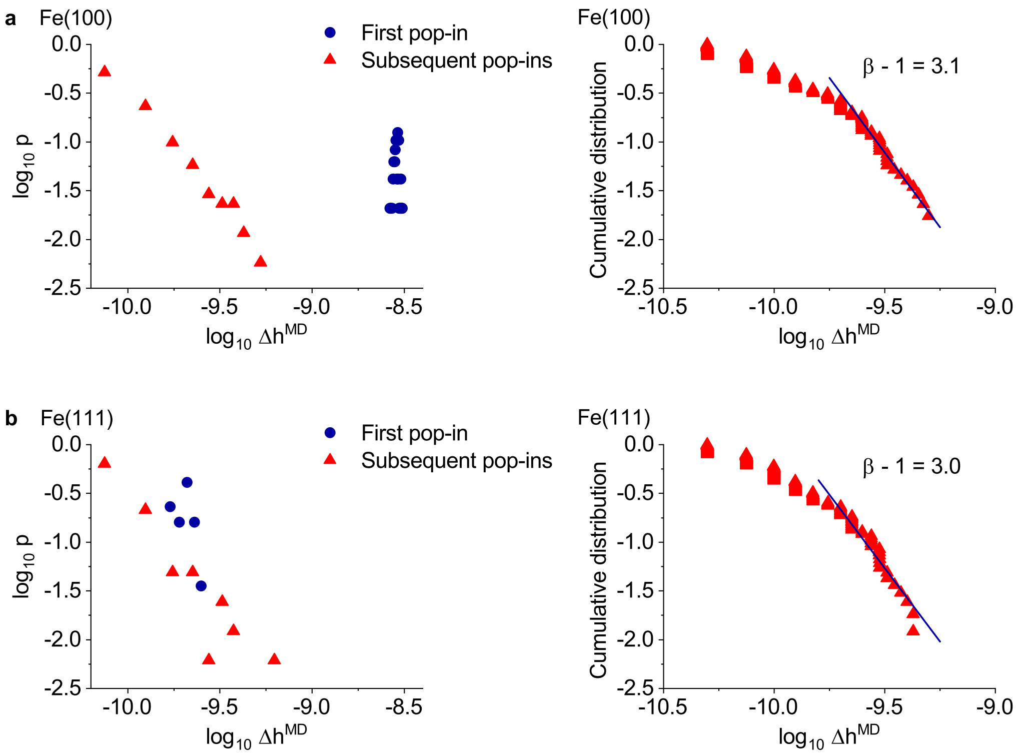

Supplementary Figure 17. Probability distributions of pop-in magnitudes as a function of a fictitious displacement burst Δh^{MD} (m) (see Supplementary Note 14) for the Fe (a) (100), (b) (111) surfaces obtained from displacement-controlled 60 MD nanoindentation simulations at 5 K (equal-width binning, 0.02 nm for first pop-in and 0.05 nm for subsequent pop-ins) giving 173 for BCC Fe (100) and 164 for BCC Fe (111) subsequent pop-ins within the statistical independent range (all of the data included other than the first force drop) (See Supplementary Figures 18 and 19). Bin-free cumulative distributions for the subsequent pop-ins are also shown in the right panel. The power law exponents were estimated by least square fitting using the data within $-9.6 \leq \log_{10}\Delta h^{\mathrm{MD}}$ for BCC Fe (100) and $-9.8 \leq \log_{10}\Delta h^{\mathrm{MD}}$ for BCC Fe (111). Please see Supplementary Note 14 for the definition of the fictitious displacement burst in displacement-controlled MD simulation. Note that because of the limited model size in MD simulation, large-scale events should be truncated, and thus power-law behavior can be seen within a limited range compared with those of the experiments.

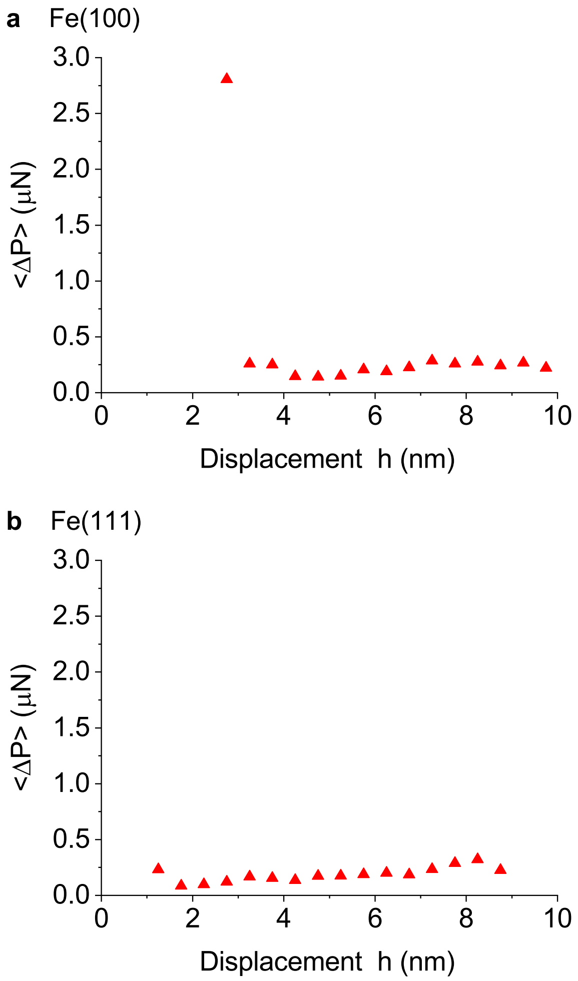

Supplementary Figure 18. Average force drop $\langle \Delta P \rangle$ vs. displacement (indentation depth) h in the MD nanoindentation simulations at 5 K on the (a) (100) and (b) (111) surfaces of BCC Fe.

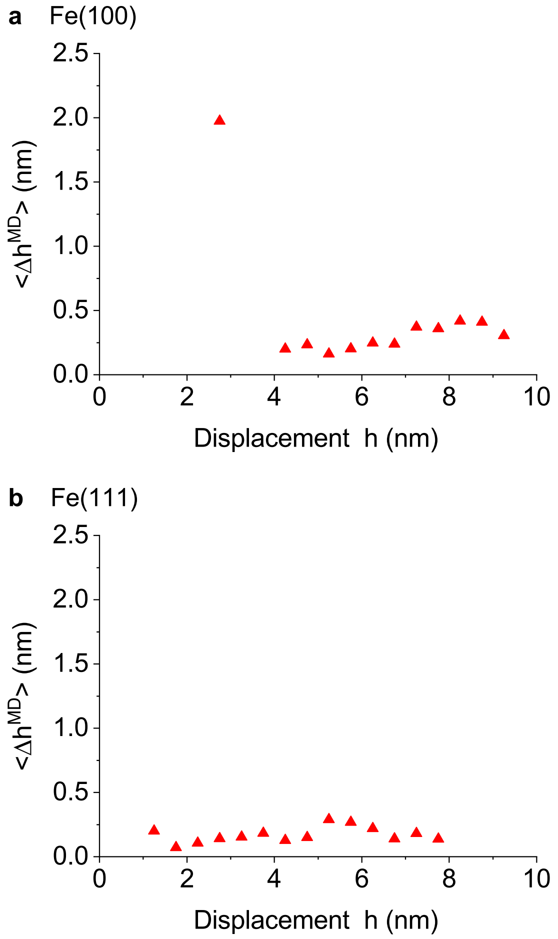

Supplementary Figure 19. Average fictitious displacement burst $\langle \Delta h^{\mathrm{MD}} \rangle$ vs. displacement (indentation depth) h in the MD nanoindentation simulations at 5 K on the (a) (100) and (b) (111) surfaces of BCC Fe.

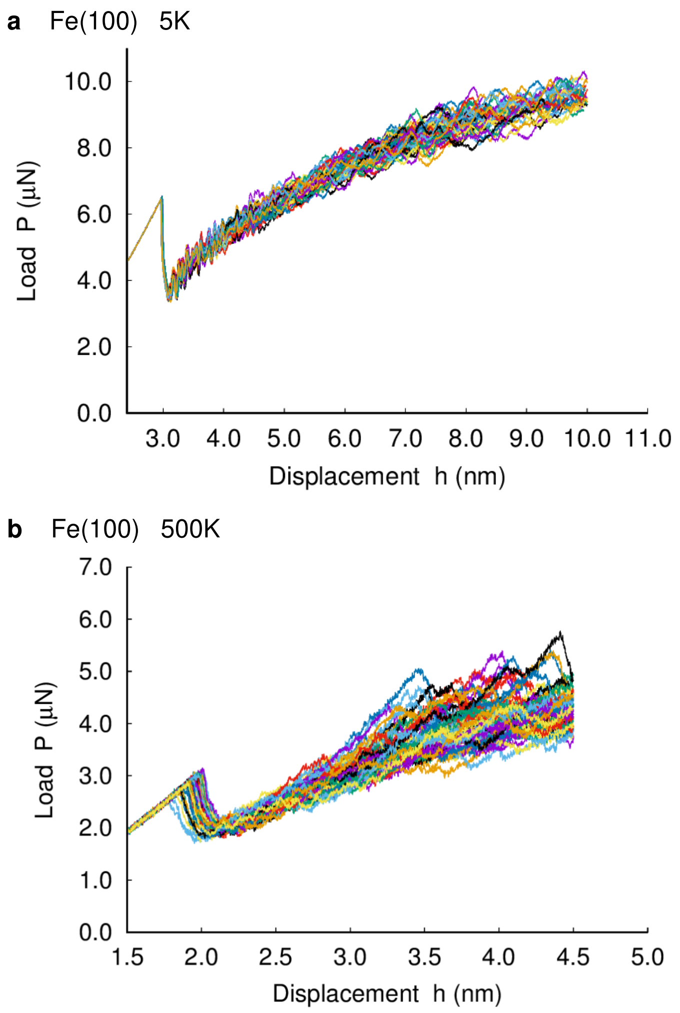

Supplementary Figure 20. Indentation load-displacement curves in the MD nanoindentation simulations on the (100) surface of the BCC Fe.

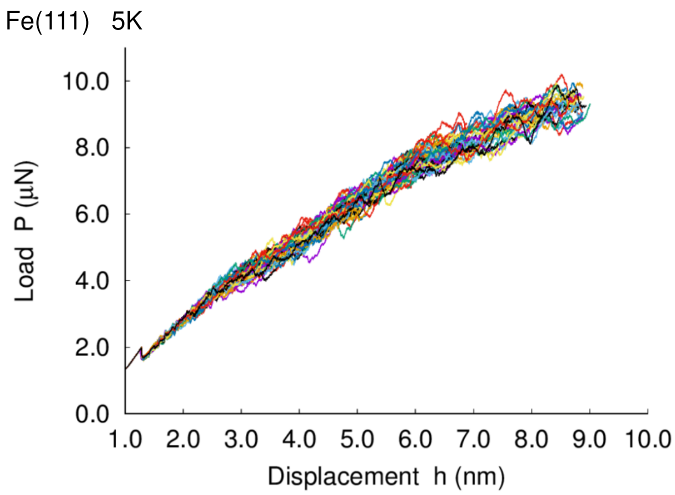

Supplementary Figure 21. Indentation load-displacement curve in the MD nanoindentation simulations on the (111) surface of the BCC Fe.

Supplementary Note 10. Average displacement burst $\langle \Delta h \rangle$ vs. displacement (indentation depth) h in nanoindentation

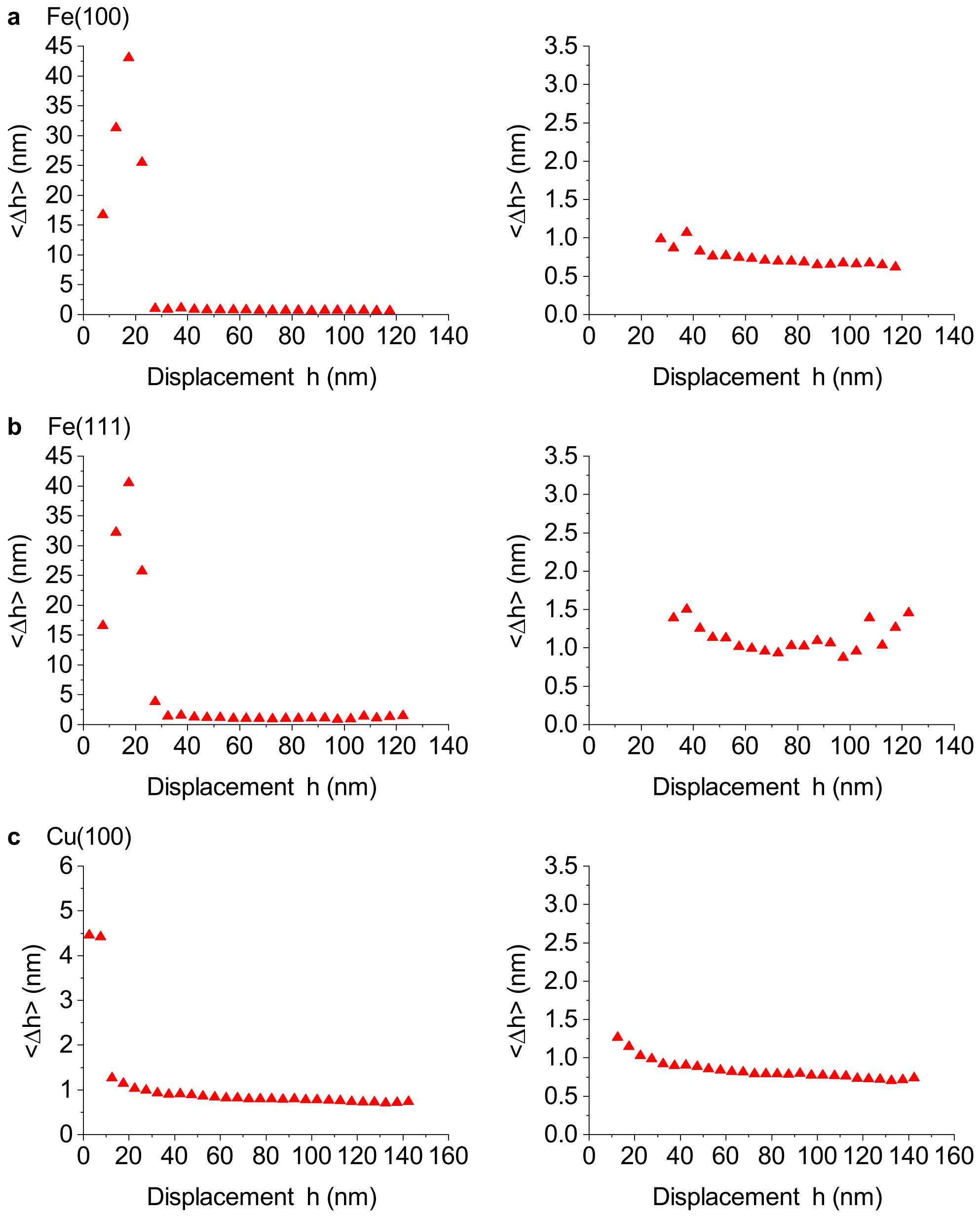

Supplementary Figure 22. Average displacement burst $\langle \Delta h \rangle$ vs. displacement (indentation depth) h in nanoindentation on the (a) (100) and (b) (111) surfaces of BCC Fe and (c) the (100) surface of FCC Cu. The right panels are magnified plot of the plot in the left panels.

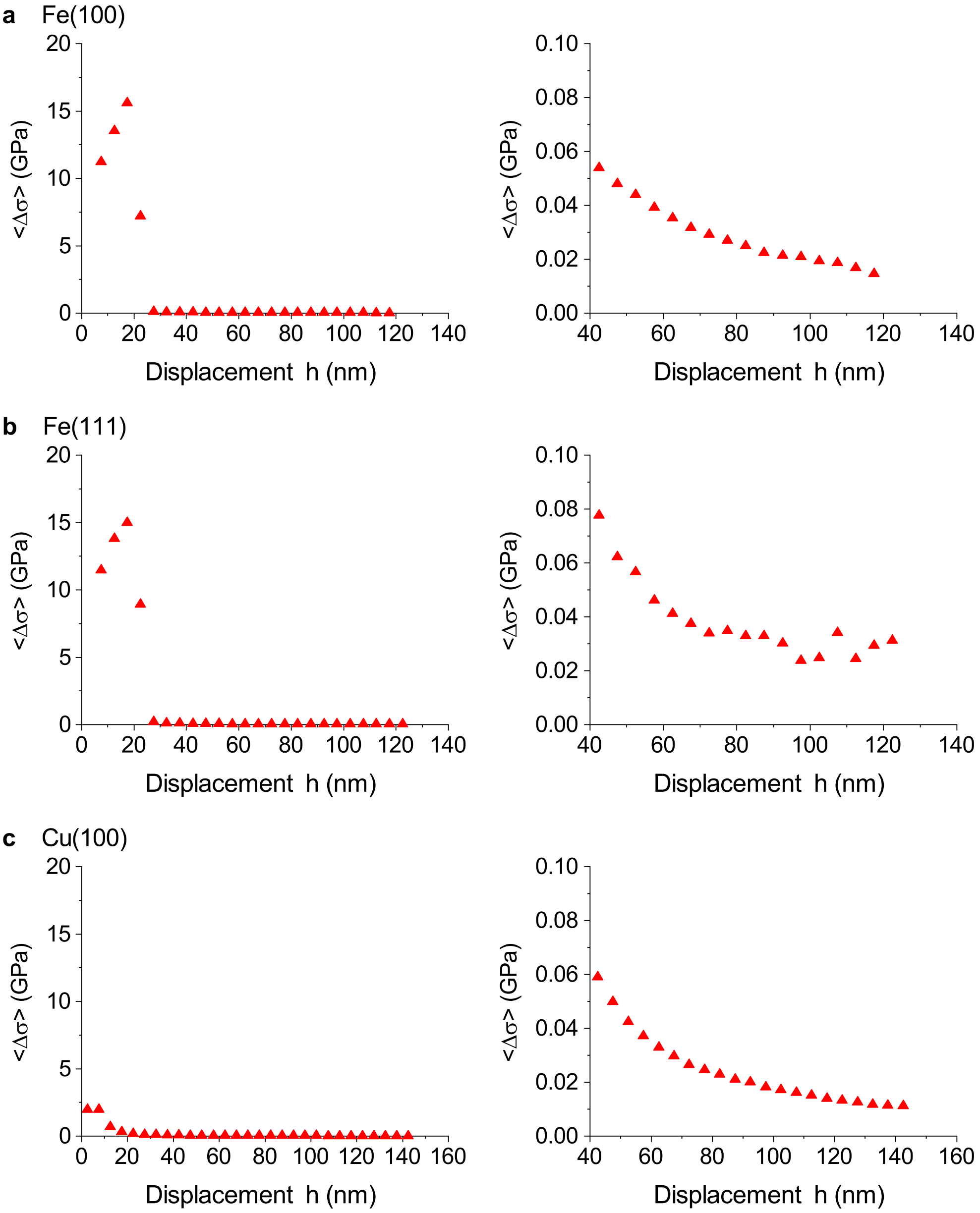

Supplementary Figure 23. Average stress drop $\langle \Delta\sigma \rangle$ vs. displacement (indentation depth) h in nanoindentation on the (a) (100) and (b) (111) surfaces of BCC Fe and (c) the (100) surface of FCC Cu. The right panels are magnified plot of the plot in the left panels.

Supplementary Note 11. Average displacement burst $\langle \Delta h \rangle$ vs. displacement (indentation depth) h in nanoindentation

- first-subsequent (all pop-in data) mixed analysis -

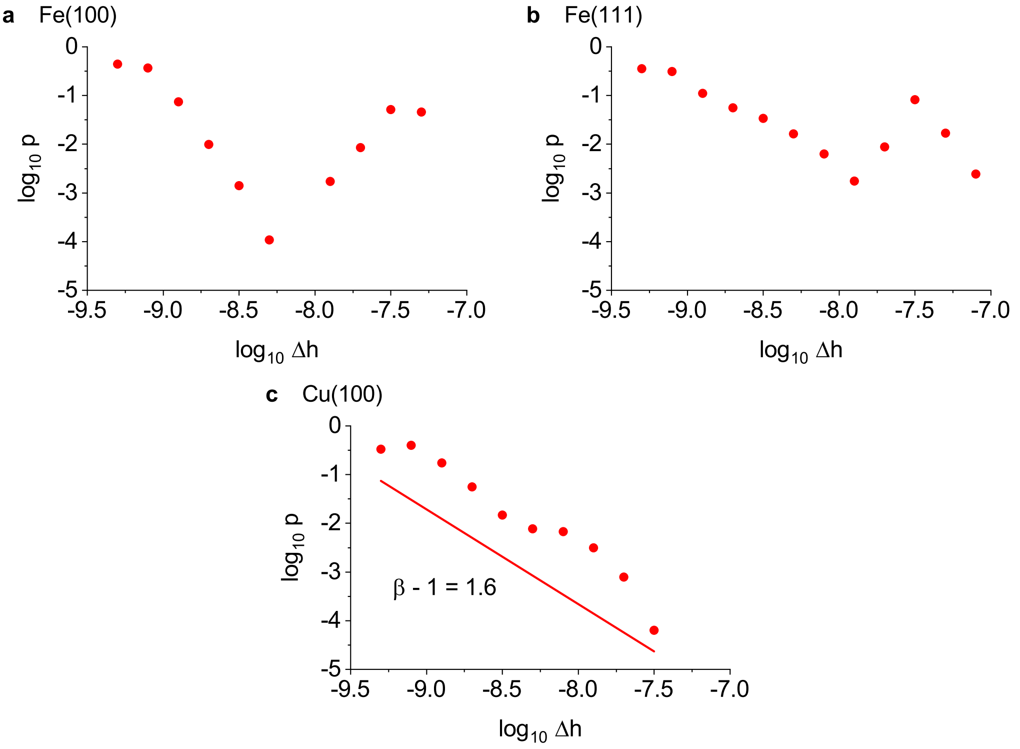

Supplementary Figure 24. Probability distributions of all pop-in magnitudes as a function of the displacement burst Δh (m) without decomposing the first and the subsequent pop-ins for the (a) (100) and (b) (111) surfaces of BCC Fe and the (c) (100) surface of FCC Cu, obtained by logarithmic binning. The solid line in (c) is a guide to the eye, represents $y \propto x^{-1.6}$.

Supplementary Note 12. Temperature dependency of power-law exponent

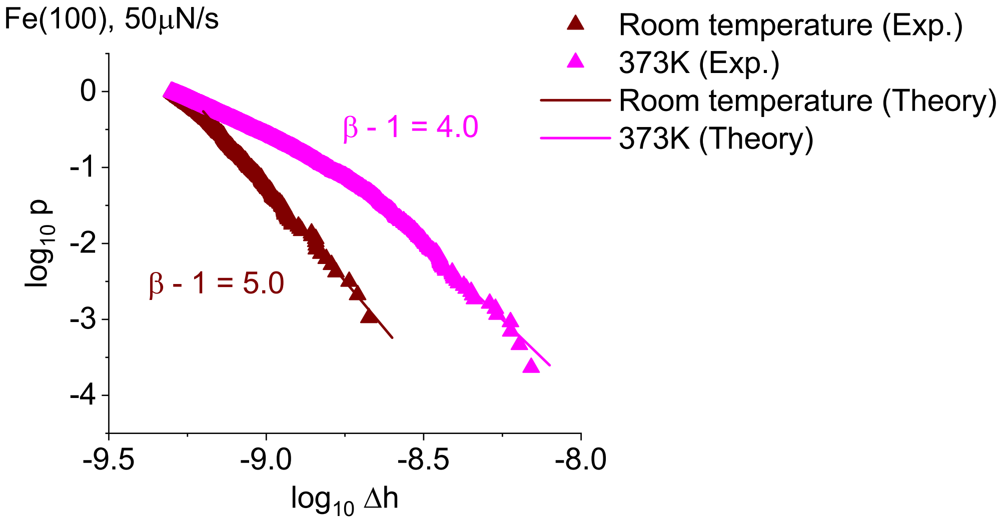

Supplementary Figure 25. Probability distribution of subsequent pop-in magnitudes as a function of the displacement burst Δh (m) for the (100) surface of BCC Fe at room temperature and 373 K with 50 μNs^{-1} loading rate, obtained by bin-free cumulative distribution. The data within the statistically independent displacement range (40.0 nm $\leq h \leq$ 120.0 nm) were used for both temperatures (see Supplementary Figures 26 and 27). The power law exponents were estimated by least square fitting using the data within -9.1 $\leq \log_{10}\Delta h$ for the room temperature and -8.7 $\leq \log_{10}\Delta h$ for 373 K. Note that these nanoindentation tests for temperature dependency analysis were performed using different nanoindentation machine from other tests. Therefore, the room temperature data (only 83 data) were different from the data shown in Figure 2 in the main text, and thus the power-law exponent is also slightly different even at the same room temperature. We also performed indentation at 473 K, but we do not show the data because a strong time-dependency (maybe dynamic strain aging) in the load-displacement curves was observed.

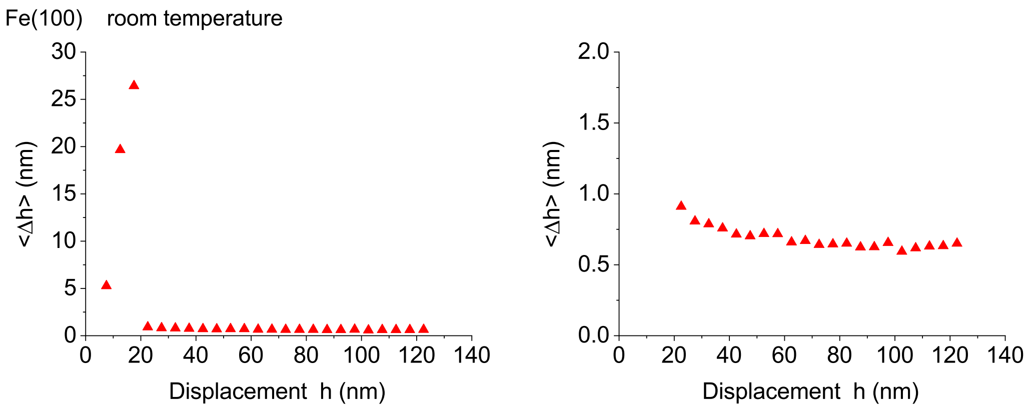

Supplementary Figure 26. Average displacement burst $\langle \Delta h \rangle$ vs. displacement (indentation depth) h in nanoindentation on the (100) surface of BCC Fe at room temperature (300 K). The right panel is magnified plot of the plot in the left panel.

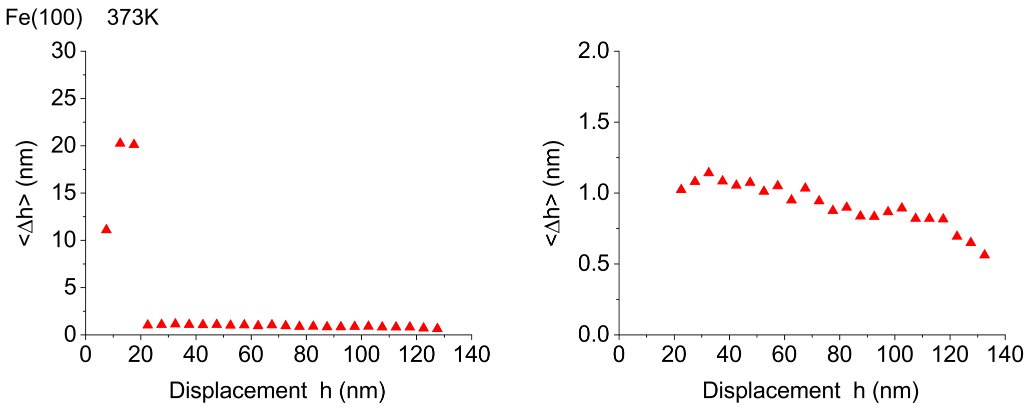

Supplementary Figure 27. Average displacement burst $\langle \Delta h \rangle$ vs. displacement (indentation depth) h in nanoindentation on the (100) surface of BCC Fe at 373 K. The right panel is magnified plot of the plot in the left panel.

Supplementary Note 13. Dislocation avalanche model based on the dislocation activities in the unique nanoindentation stress field

- Theory of power law distribution for the pop-in distance -

Close observation on molecular dynamics simulation (Figure 4 in the main text and Supplementary Figures 31 and 32) indicates that the size of subsequent pop-ins is determined by the total moving distance of mobile dislocations. While each dislocation moves different distance during a single pop-in event, here we adopt a mean-field formulation and focus on the average moving distance denoted by L. The average moving distance may vary for each pop-in event, and this fluctuation leads to the power-law distribution for the pop-in distance. In the following, we derive the probability distribution of L.

Dislocations are driven by the stress τ, which consists of the background stress τ_0 and the backstress caused by the interaction between dislocations.

$$\tau \simeq \tau_0 - \frac{\tau_{\mathrm{int}} b}{r}, \tag{S24}$$

where the second term is the effective interaction stress as a function of the average distance between dislocations, r. Additionally, τ_{int} denotes an effective stress constant. For the stress field caused by a single dislocation, τ_{int} is approximately the shear modulus. However, here we consider the mean-field stress caused by many dislocations. Since it should be a superposition of attractive and repulsive contributions, τ_{int} may be much smaller than the shear modulus. However, the precise derivation of τ_{int} is not feasible at present, and therefore Eq. (S24) should be interpreted as a phenomenological mean-field model.

In the indentation geometry, dislocations move radially from the proximity of indenter to the interior of the specimen. Therefore, dislocations move away from each other if they are gliding on non-parallel slip planes. As a result, the average distance between dislocations increases with their motion. For instance, the distance between two dislocations on different slip planes that intersect with angle θ should increase by $l \sin(\theta/2)$ if each dislocation moves the distance of l keeping the common depth. More generally, we may assume that $r \simeq r_0 + gl$, where l is the average moving distance of dislocations during a pop-in event, g is

a geometrical factor, and r_0 is the initial average distance at the onset of an event (when $l = 0$). Then Eq. (S24) becomes

$$\tau \simeq \tau_0 - \frac{\tau_{\rm int} b}{r_0 + gl}. \tag{S25}$$

Note that l increases from 0 to L during a single pop-in event and the interaction stress is $-\tau_{\rm int} b/r_0$ at the onset of a pop-in event.

Since the motion of dislocations is a thermal activation process, the probability that the moving distance of a dislocation segment increases from l to $l+b$ is given as

$$P(l+b|l) \propto \exp\left(\frac{\tau\Omega}{kT}\right). \tag{S26}$$

Here Ω is the activation volume that may depend on the temperature and the stress. Inserting Eq. (S25) to Eq. (S26), one obtains

$$P(l+b|l) \propto \exp\left[-\frac{\tau_{\rm int}\Omega}{kT}\frac{b}{r_0+gl}\right]. \tag{S27}$$

The probability of realizing the moving distance larger than L is given as

$$P_>(L) = \prod_{l=0}^{L} P(l+b|l) \propto \exp\left(-\frac{\tau_{\rm int}\Omega}{kT}\sum_{l=0}^{L}\frac{b}{r_0+gl}\right), \tag{S28}$$

where $l = 0, b, 2b, \cdots, L$. Replacing the summation with an integral, one is led to the power law distribution for L.

$$P_>(L) \simeq \exp\left(-\frac{\tau_{\rm int}\Omega}{kT}\int_0^L \frac{dl}{r_0+gl}\right) = \exp\left[-\frac{\tau_{\rm int}\Omega}{gkT}\log\left(1+\frac{gL}{r_0}\right)\right] \sim L^{-\beta}, \tag{S29}$$

where the exponent $\beta = \tau_{\rm int}\Omega/gkT$.

The probability distribution for L is actually the same as that for the pop-in distance, Δh. We may assume that Δh is proportional to the plastic strain accommodated in the region beneath the indenter. The Orowan's relation suggests that the plastic strain is proportional to the moving distance of dislocations, L. Thus, L is proportional to Δh, leading to the common power-law distribution for these

two quantities.

The derivation here implies that the exponent β is not universal. Particularly, the exponent should decrease for higher temperature. The exponent also depends on the geometrical factor g and the activation volume Ω, and therefore it should depend on the material and the crystalline orientation. These tendencies are confirmed in our experiments with different loading rates (Supplementary Note 12), partly supporting our model. However, estimate of the absolute value of the exponent is not straightforward, since τ_{int} is not derived in terms of many dislocation systems.

Next we explain the difference in the exponents for two types of testing: indentation and pillar compression. In pillar compression tests, the amplitude of axial strain jump obeys the power-law distribution with the exponent around 1.5, whereas much larger exponent is obtained in our indentation test. The difference may be attributed to the relation between the moving distance l and the mean spacing of dislocations r. In indentation tests, $r = r_0 + gl$, which results from the radial (or conical) propagation pattern of dislocations. In pillar compression, however, the motion of dislocations is not radial and therefore the above relation does not hold. Rather, the mean dislocation spacing undergoes large fluctuation. Dislocations pile up in a dislocation band and work as a floodgate, which gives way to swarming dislocations, leading to an avalanche [18]. Therefore, fluctuation in the mean spacing r should be taken into account in the case of pillar compression. Here we write $r(l) = r_0[1+\xi(l)]$, where $r_0\xi(l)$ denotes the fluctuation in r. Using this relation, Eq. (S24) becomes

$$\tau(l) \simeq \tau_0 - \frac{\tau_{\text{int}} b}{r_0} \frac{1}{1+\xi(l)}. \tag{S30}$$

Namely, the motion of dislocation is subjected to the stress fluctuation in addition to thermal fluctuation. Supposing that $r_0 \sim 10^{-8}$ m (corresponding to the dislocation density of 10^{16} m^{-2}), and that τ_{int} is an order of magnitude smaller than the shear modulus, the amplitude of stress fluctuation ($\tau_{\text{int}} b/r_0$) is on the order of 10^7 Pa. Since this is several orders of magnitude larger than thermal fluctuation in stress, kT/b^3, the motion of dislocations should be dominated by the athermal stress fluctuation caused by $\xi(l)$. Thus, avalanche dynamics is rather athermal in pillar compression tests, whereas it is thermal in indentation tests. As is explained

above, the difference is due to the vital role of dislocation pileups in causing dislocation avalanches in pillar compression, whereas such a mechanism is absent in indentation.

Taking the above discussion into account, let us assume that an avalanche starts at a certain threshold stress denoted by τ_s, and ends if the stress drops below another threshold, τ_r. Then the fluctuating stress $\tau(l)$ must satisfy the following conditions: $\tau(0) = \tau_s$, $\tau(l) > \tau_r$ for $0 \leq l < L$, and $\tau(L) = \tau_r$. To derive the probability distribution of L under this condition is known as the first-passage problem, and the solutions are already given for various cases [19]. For instance, if the fluctuation in the dislocation spacing $\xi(l)$ is memoryless or has a short (exponential) memory, the distribution function of L has the tail proportional to $L^{-1.5}$. More generally, if $\xi(l)$ has the long-term memory characterized by the Hurst exponent H, the distribution function of L has the tail of L^{-1-H} [20]. For instance, the exponent of $5/3$, which appears to be another accepted value for avalanche distribution, corresponds to $H = 2/3$. This may result from a long-range correlation in the spacing of dislocations, which affects the nature of fluctuation in $\xi(l)$.

Lastly, we wish to remark that the exponent of 1.5 is explained based on another type of mean-field model [21]. Although the model is different from the present one, the athermal nature of dislocation motion and their interactions are somewhat common to the above settings.

Supplementary Note 14. Algorithm of load drop sampling from load displacement curve given by displacement-controlled MD nanoindentation simulation and definition of "fictitious" displacement burst

Algorithm of load drop sampling

After displacement control MD simulation, we have MD load-displacement sequential data $P(h_i)$ with $h_{i+1} - h_i = 2.5 \times 10^{-2}$ nm displacement mesh ($0 \leq i \leq N_{\text{data}}$). Initially, we set the load drop $P_{\text{drop}} = 0$. Checking the data from small i to large i sequentially, if $P(h_{i-1}) < P(h_i)$ $(i \geq 1)$, then $P_{\text{drop}} = P_{\text{drop}} + (P(h_i) - P(h_{i-1})$. If $P(h_{i-1}) > P(h_i)$ $(i \geq 1)$ and $P_{\text{drop}} > \Delta P_1^{\text{threshold}}$, then supposing a pop-in event occurred and thus a continuous load drop was finished. Then, we recorded the P_{drop} as one of the "pop-in load drop". The threshold $\Delta P_1^{\text{threshold}}$ was set to 0.05 μN. Note that for a displacement-controlled simulation, the definition of avalanche events should be equivalent to the force drops [16]. Although the aforementioned definition are not formally the events because the defined displacements are force-balanced by applied loads, the deviation between the displacements and ones which are truly equivalent to the force drops should be always constant, while targeting the same nanoindentation system. Therefore, at least the outlines of pop-in distributions can be captured even by the definition.

Definition of "fictitious" displacement burst in displacement-controlled MD simulation

Concerning estimation of the displacement burst Δh, in the displacement-controlled MD simulation, the indentation load drops immediately after each pop-in. As the indenter is displaced further, the load increases again and eventually reaches the level where the pop-in started, as shown in Figures 3(f) and 4(f) in the main text. The load recovered point was assumed to be corresponding to the end of the pop-in. The displacement from the load drop point to the load recovered point was defined as fictitious displacement burst (see Supplementary Figure 28). Thus, the next pop-in was defined as the point at which the indentation load dropped firstly after the end of the last pop-in, which can be detected using the algorithm of load drop sampling. Thus, once we detected the "pop-in load drop" in above algo-

rithm, we searched a following displacement data point $i^{\mathrm{after-pop-in}}$ after the load drop, which satisfies condition, $P(h_{i^{\mathrm{before-pop-in}}}) = P(h_{i^{\mathrm{after-pop-in}}})$ (load recovering) is within a error below $\Delta P_2^{\mathrm{threshold}}$, where $i^{\mathrm{before-pop-in}}$ is the last displacement data point having $P_{\mathrm{drop}} = 0$ immediately before the load drop. Then, the displacement burst can be computed by $\Delta h^{\mathrm{MD}} \equiv h_{i^{\mathrm{after-pop-in}}} - h_{i^{\mathrm{before-pop-in}}}$. The threshold $\Delta P_2^{\mathrm{threshold}}$ was set to 0.03 μN.

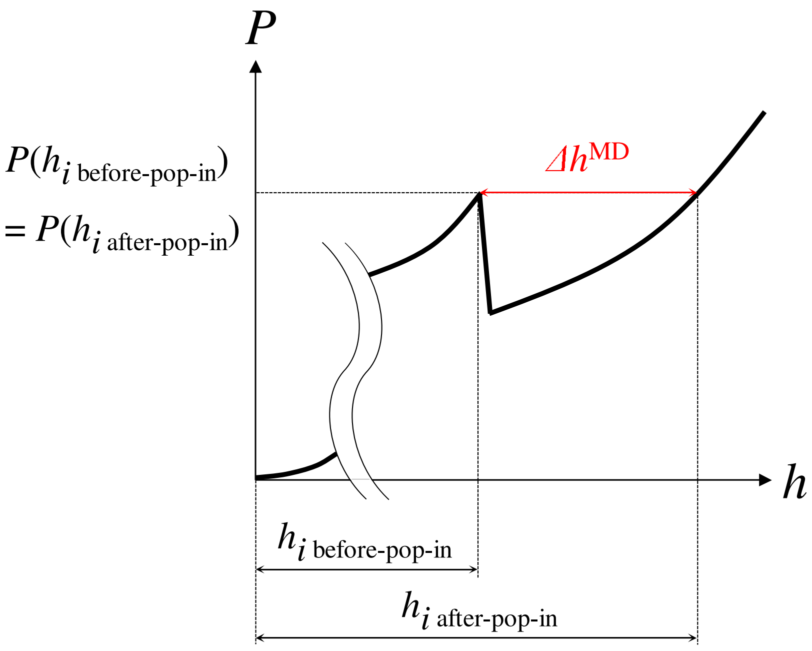

Supplementary Figure 28. Schematic illustration of fictitious displacement burst Δh^{MD}.

Supplementary Note 15. Visualizations of 1) defect structure and 2) von Mises stress distribution beneath the indenter immediately before and after the first pop-in, and distribution of 3) von Mises atomic-strain invariant and 4) atomic displacement along indenter axis during the first pop-in

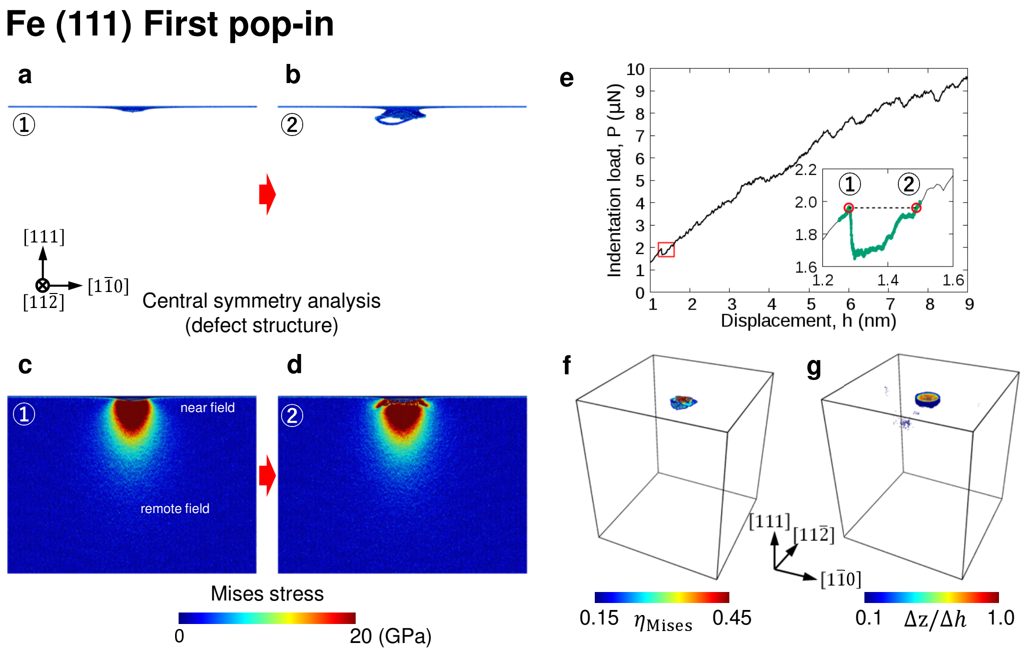

Supplementary Figure 29. First pop-in behavior in molecular dynamics simulation at 5 K on the (111) BCC Fe surface. Defect structure immediately (a) before and (b) after the first pop-in (central symmetry parameter coloring [17]), von Mises stress distribution immediately (c) before and (d) after the first pop-in, and (e) corresponding load-displacement curve. (f) Spatial distribution of von Mises atomic-strain invariant, η_{Mises}. Only atoms satisfying $\eta_{\text{Mises}} > 0.15$ are displayed. (g) Spatial distribution of atomic displacement along loading direction ($[111]$), Δz, normalized by displacement burst Δh. Only atoms satisfying $\Delta z/\Delta h > 0.1$ are displayed. The von Mises stress distribution is shown on a $(11\bar{2})$ plane passing through the indenter central axis. The movies of the dislocation pattern and the stress distribution evolution during the simulation can be found in Supplementary Movie 1.

Cu (100) First pop-in

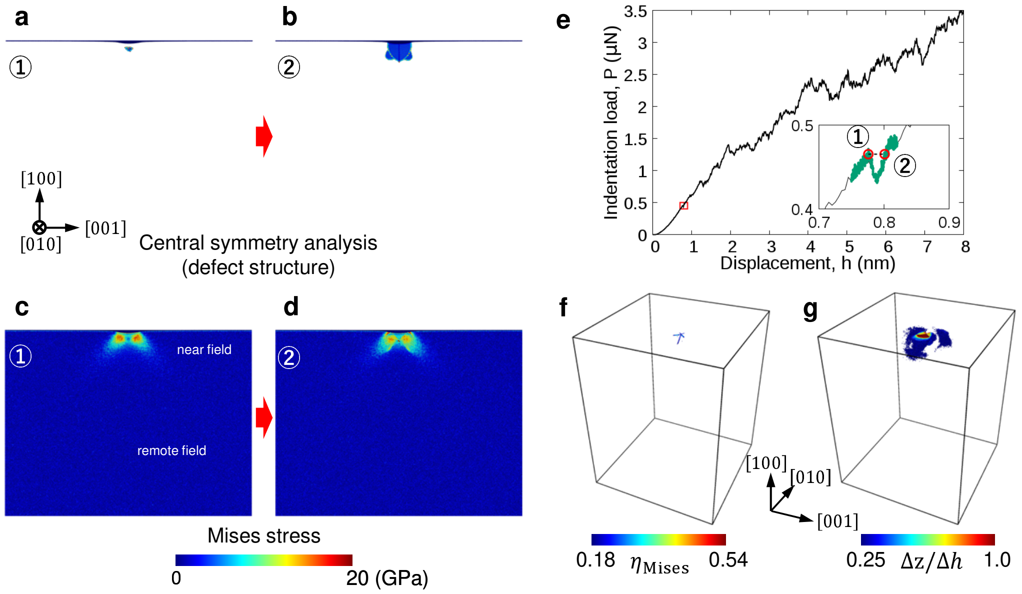

Supplementary Figure 30. First pop-in behavior in molecular dynamics simulation at 5 K on the (100) FCC Cu surface. Defect structure immediately (a) before and (b) after the first pop-in (central symmetry parameter coloring [17]), von Mises stress distribution immediately (c) before and (d) after the first pop-in, and (e) corresponding load-displacement curve. (f) Spatial distribution of von Mises atomic-strain invariant, η_{Mises}. Only atoms satisfying $\eta_{\text{Mises}} > 0.18$ are displayed. (g) Spatial distribution of atomic displacement along loading direction ([100]), Δz, normalized by displacement burst Δh. Only atoms satisfying $\Delta z/\Delta h > 0.25$ are displayed. The von Mises stress distribution is shown on a (010) plane passing through the indenter central axis. The movies of the dislocation pattern and the stress distribution evolution during the simulation can be found in Supplementary Movie 1.

Supplementary Note 16. Visualizations of 1) defect structure and 2) von Mises stress distribution beneath the indenter immediately before and after a subsequent pop-in, and distribution of 3) von Mises atomic-strain invariant and 4) atomic displacement along indenter axis during the subsequent pop-in

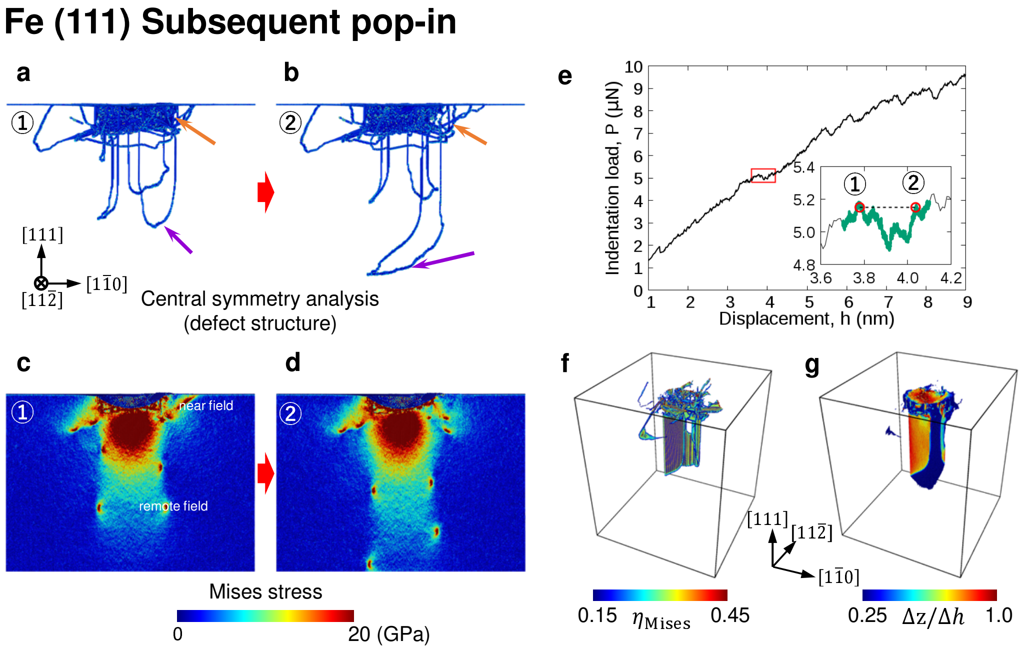

Supplementary Figure 31. Subsequent pop-in behavior in molecular dynamics simulation at 5 K on the (111) BCC Fe surface. Defect structure immediately (a) before and (b) after a subsequent pop-in (central symmetry parameter coloring [17]), von Mises stress distribution immediately (c) before and (d) after a subsequent pop-in, and (e) corresponding load-displacement curve. (f) Spatial distribution of von Mises atomic-strain invariant, η_{Mises}. Only atoms satisfying $\eta_{\text{Mises}} > 0.15$ are displayed. (g) Spatial distribution of atomic displacement along loading direction ($[111]$), Δz, normalized by displacement burst Δh. Only atoms satisfying $\Delta z/\Delta h > 0.25$ are displayed. The von Mises stress distribution is shown on a $(11\bar{2})$ plane passing through the indenter central axis. The movies of the dislocation pattern and the stress distribution evolution during the simulation can be found in Supplementary Movie 1.

Cu (100) Subsequent pop-in

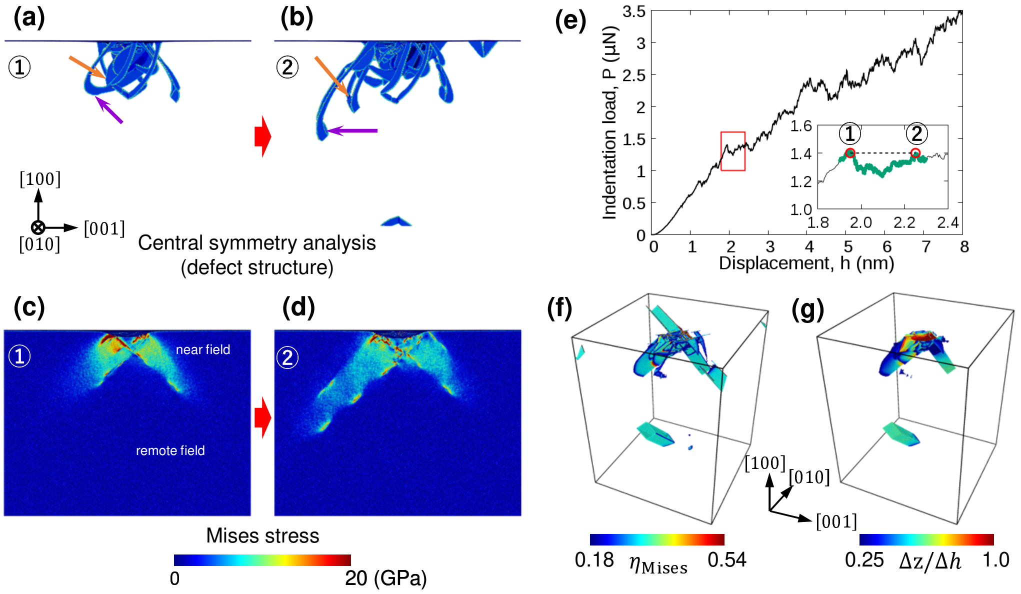

Supplementary Figure 32. Subsequent pop-in behavior in molecular dynamics simulation at 5 K on the (100) FCC Cu surface. Defect structure immediately (a) before and (b) after a subsequent pop-in (central symmetry parameter coloring [17]), von Mises stress distribution immediately (c) before and (d) after a subsequent pop-in, and (e) corresponding load-displacement curve. (f) Spatial distribution of von Mises atomic-strain invariant, η_{Mises}. Only atoms satisfying $\eta_{\mathrm{Mises}} > 0.18$ are displayed. (g) Spatial distribution of atomic displacement along loading direction ([100]), Δz, normalized by displacement burst Δh. Only atoms satisfying $\Delta z/\Delta h > 0.25$ are displayed. The von Mises stress distribution is shown on a (010) plane passing through the indenter central axis. The movies of the dislocation pattern and the stress distribution evolution during the simulation can be found in Supplementary Movie 1.

Supplementary Note 17. Typical thickness of the oxide layer forming on the surface of tested BCC Fe samples

To investigate the oxide layer forming on the surface of the Fe, we measured the thickness of the oxide layer by using a spectroscopic ellipsometer (MARY-102FM, Five Lab, Yokohama, Japan). The machine was operated in Rotating Retarder Method by using 0.8 mW HeNeLaser of 0.8 mm in diameter with a single wave length of 632.8 nm. The incident beam angle was set as 69.97° and the reflected beam angle was measured with the resolution of $\pm 0.01°$. The beam scanned the sample surface in the area of 1×1 mm^2 with 0.25 mm step resulting in 5×5 positions. We assumed an oxide as Fe_2O_3 or Fe_3O_4 with refractive index of 2.918 or 2.42, respectively, to measure a thickness of the oxide layer. The 2D map of the measured thickness of the Fe_2O_3 layer are shown in the following Supplementary Figure 33. The discrete data are connected with a spline curve in between with 10 pixels resolution, resulting 40×40 pixels^2. The map shows a gradual distribution in thickness between the max. 6.15 nm and min. 4.98 nm, and the average thickness was found to be about 5.49 nm. We conclude that this thickness is within the negligible range compared to the radius of the indenter R ($\sim$ 500 nm). Since the oxide could be much harder than iron, the oxide acts as a "pseudo" indenter tip to iron when a load is applied by a "real" diamond indenter to the surface oxide. Based on Hertz's contact theory, a maximum shear stress underneath the indenter is inversely proportional to $R^{\frac{2}{3}}$, and 1 % deviation in R corresponds to 0.67 % difference in the shear stress, which is absolutely within an experimental error.

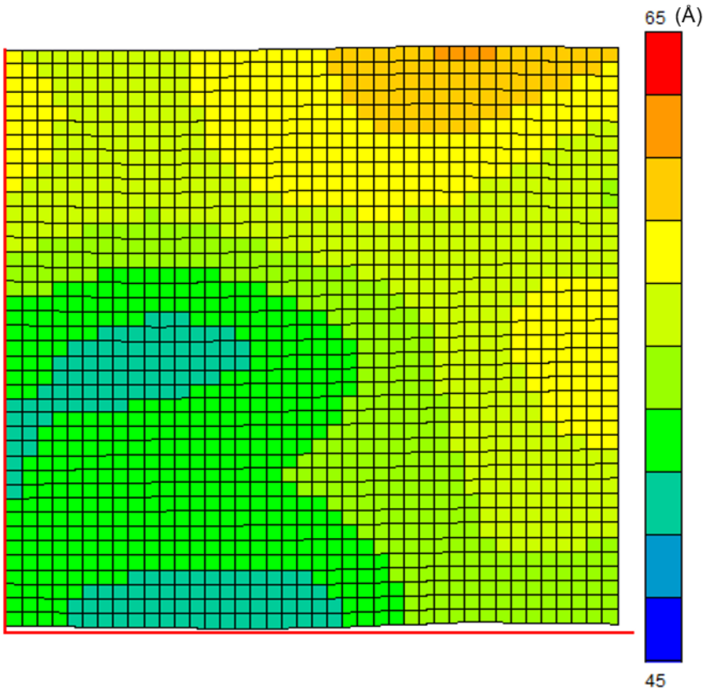

Supplementary Figure 33. The 2D map of the thickness of the oxide layer in the area 1×1 mm^2 on the sample surface of electropolished Fe (001) sample. The minimum and maximum thicknesses are 4.98 nm and 6.15 nm, respectively, and the average for the whole area is 5.49 nm, which is about 1 % of the curvature radius of the indenter and negligible.

Supplementary Note 18. Typical atomic force microscope (AFM) image of BCC Fe surface

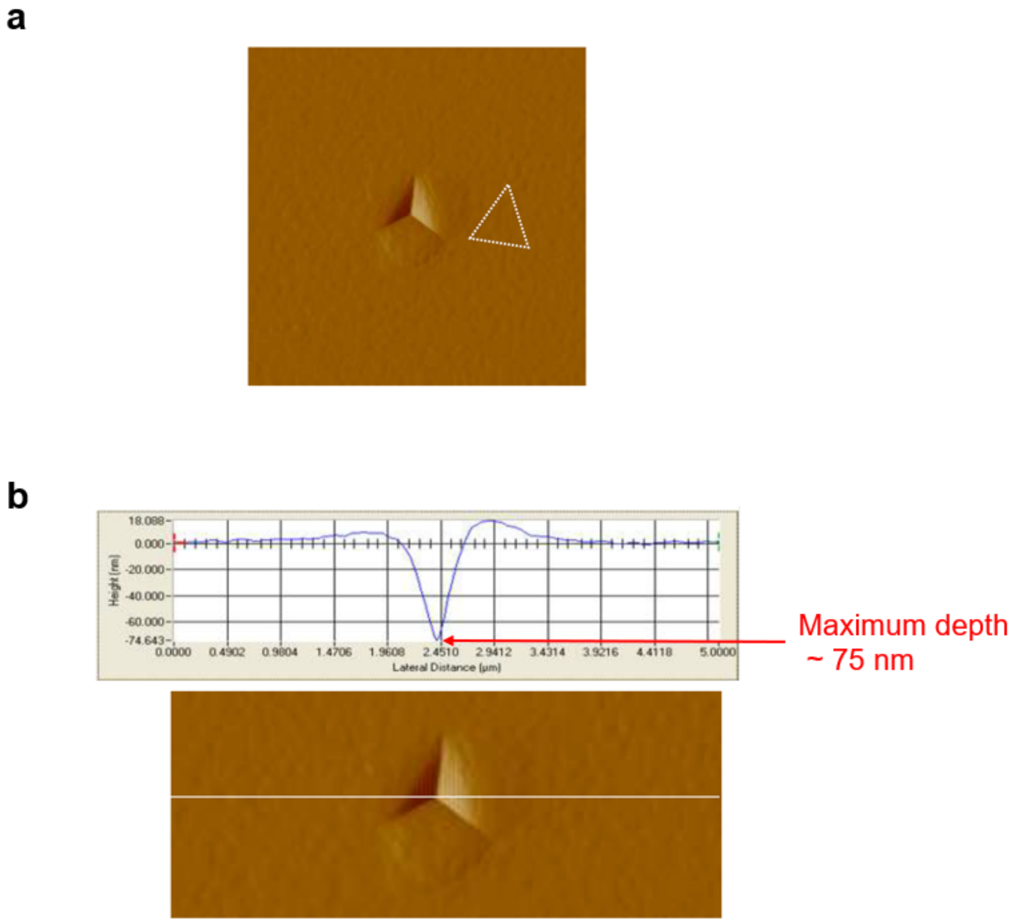

Supplementary Figure 34. (a) Atomic force microscope (AFM) image of the (100) surface of the BCC Fe sample with an indent. The scan area was 5×5 μm^2. (b) Line-profile of the AFM image. The identified maximum depth is about 75 nm, which is shallower than 100 nm.

Supplementary Note 19. Load-displacement curves used for pop-in magnitude sampling

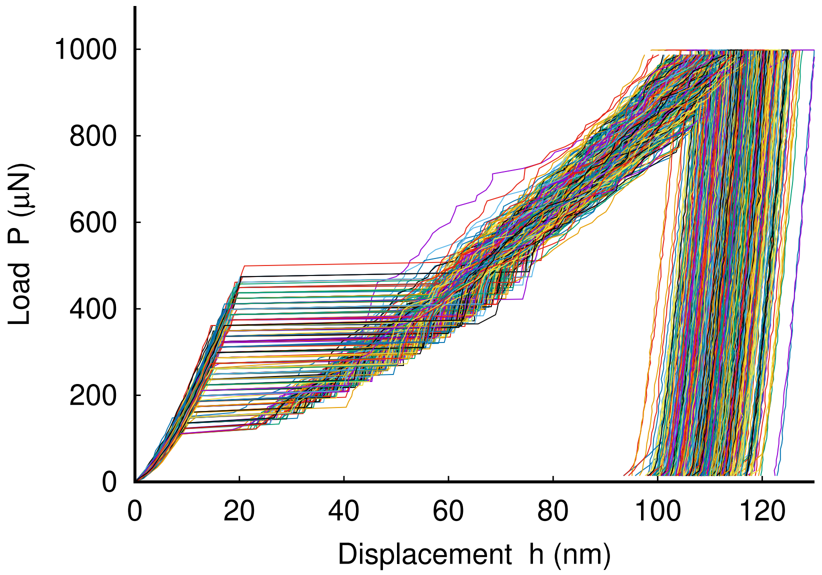

Supplementary Figure 35. Indentation load-displacement curves in 1000 nanoindentations on the (100) surface of the BCC Fe.

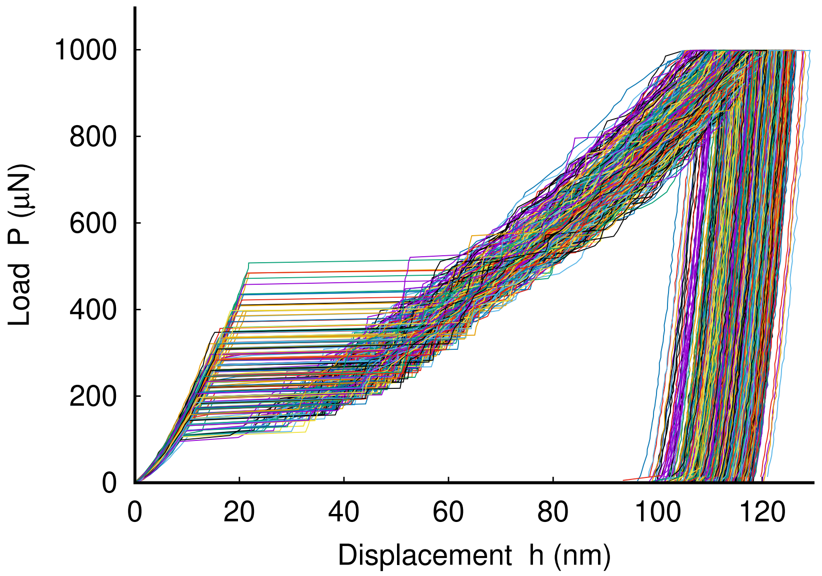

Supplementary Figure 36. Indentation load-displacement curves in 1000 nanoindentations on the (111) surface of the BCC Fe.

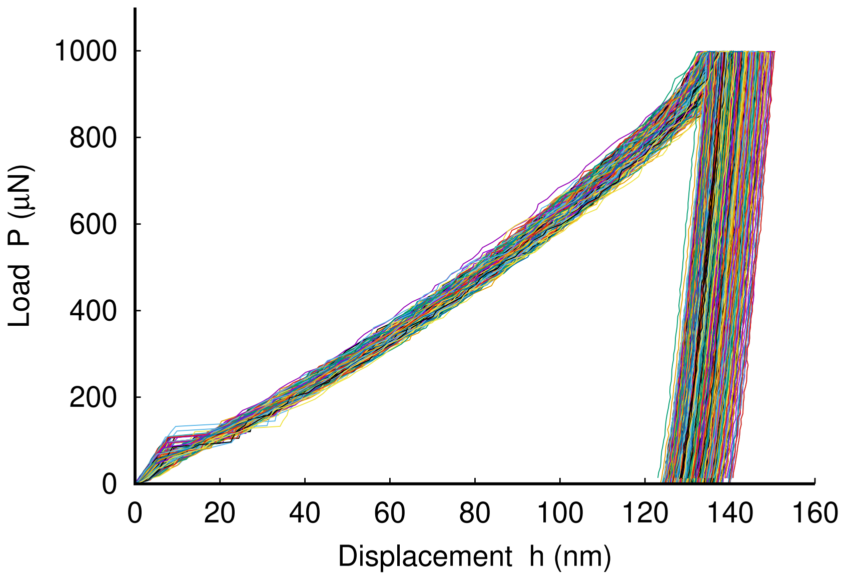

Supplementary Figure 37. Indentation load-displacement curves in 1000 nanoindentations on the (100) surface of the FCC Cu.

Supplementary References

[1] Johnson, K. L. *Contact Mechanics* (Cambridge University Press, Cambridge, 1985).

[2] Oliver, W. C. & Pharr, G. M. An improved technique for determining hardness and elastic modulus using load and displacement sensing indentation experiments. *J. Mater. Res.* **7**, 1564–1583 (1992).

[3] Bykov, M. *et al.* High-pressure synthesis of ultraincompressible hard rhenium nitride pernitride $Re_2(N_2)(N)_2$ stable at ambient condition. *Nature Comm.* **10**, 2994 (2019).

[4] Kim, Y.-Y. *et al.* Tuning hardness in calcite by incorporation of amino acids. *Nature Mater.* **15**, 903–910 (2016).

[5] Ketov, S. V. *et al.* Rejuvenation of metallic glasses by non-affine thermal strain. *Nature* **524**, 200–203 (2015).

[6] Mei, Y. *et al.* Combinatorial development of biomaterials for clonal growth of human pluripotent stem cells. *Nature Mater.* **9**, 768–778 (2010).

[7] Suresh, S. Graded materials for resistance to contact deformation and damage. *Science* **292**, 2447–2451 (2001).

[8] Sato, Y., Shinzato, S., Ohmura, T. & Ogata, S. Atomistic prediction of the temperature- and loading-rate-dependent first pop-in load in nanoindentation. *Int. J. Plast.* **121**, 280–292 (2019).

[9] Fan, Y., Osetsky, Y. N., Yip, S. & Yildiz, B. Onset mechanism of strain-rate-induced flow stress upturn. *Phys. Rev. Lett.* **109**, 135503 (2012).

[10] Zhu, T., Li, J., Samanta, A., Leach, A. & Gall, K. Temperature and strain-rate dependence of surface dislocation nucleation. *Phys. Rev. Lett.* **100**, 025502 (2008).

[11] Kocks, U. F., Argon, M. F. & Ashby, M. F. *Thermodynamics and kinetics of slip* (Pergamon Press, Oxford, 1975).

[12] Li, T. L., Gao, Y. F., Bei, H. & George, E. P. Indentation Schmid factor and orientation dependence of nanoindentation pop-in behavior of NiAl single crystals. *J. Mech. Phys. Solids* **59**, 1147–1162 (2011).

[13] Dasai, P. D. Thermodynamic properties of iron and silicon. *J. Phys. Chem. Pef. Data* **15**, 967–983 (1986).

[14] Smithels, C. I. *Metals Reference Book* (Butterworths, London, 1976).

[15] Ruzic, J., Watanabe, I., Goto, K. & Ohmura, T. Nano-Indentation Measurement for Heat Resistant Alloys at Elevated Temperatures in Inert Atmosphere. *Mater. Trans.* **60**, 1411–1415 (2019).

[16] Song, H., Yavas, H., van der Giessen, E. & Papanikolaou, S. Discrete dislocation dynamics simulations of nanoindentation with pre-stress: Hardness and statistics of abrupt plastic events. *J. Mech. Phys. Solids* **123**, 332–347 (2019).

[17] Kelchner, C. L., Plimpton, S. J. & Hamilton, J. C. Dislocation nucleation and defect structure during surface indentation. *Phys. Rev. B* **58**, 11085–11088 (1998).

[18] Hu, Y., Shu, L., Yang, Q. *et al.* Dislocation avalanche mechanism in slowly compressed high entropy alloy nanopillars. *Commun. Phys.* **1**, 61 (2018).

[19] Metzler, R. & Klafter, J. The Restaurant at the End of the Random Walk: Recent Developments in Fractional Dynamics of Anomalous Transport Processes. *J. Phys. A* **37**, R161–R208 (2004).

[20] Rangarajan, G. & Ding, M. First passage time distribution for anomalous diffusion. *Phys. Lett. A* **273**, 322–330 (2000).

[21] Dahmen, K. A., Ben-zion, Y. & Uhl, J. T. Micromechanical Model for Deformation in Solids with Universal Predictions for Stress-Strain Curves and Slip Avalanches. *Phys. Rev. Lett.* **102**, 175501 (2009).
