# Supplementary material for: Unique universal scaling in nanoindentation pop-ins
Source: Nat Commun. 2020 Aug 21;11:4177. doi: 10.1038/s41467-020-17918-7 (PMC7443148; doi:10.1038/s41467-020-17918-7)
Supplement: Supplementary file 3 — Description of Additional Supplementary Files [file 41467_2020_17918_MOESM3_ESM.docx]

Description of Additional Supplementary Files

Title: Supplementary Movie 1

Description: Movies of the dislocation pattern and stress distribution evolution during nanoindentation MD simulations for the (100) and (111) BCC Fe surfaces and the (100) FCC Cu surface
