# Supplementary figures and images for: Unique universal scaling in nanoindentation pop-ins
Source: Nat Commun. 2020 Aug 21;11:4177. doi: 10.1038/s41467-020-17918-7 (PMC7443148; doi:10.1038/s41467-020-17918-7)

## Slide 1
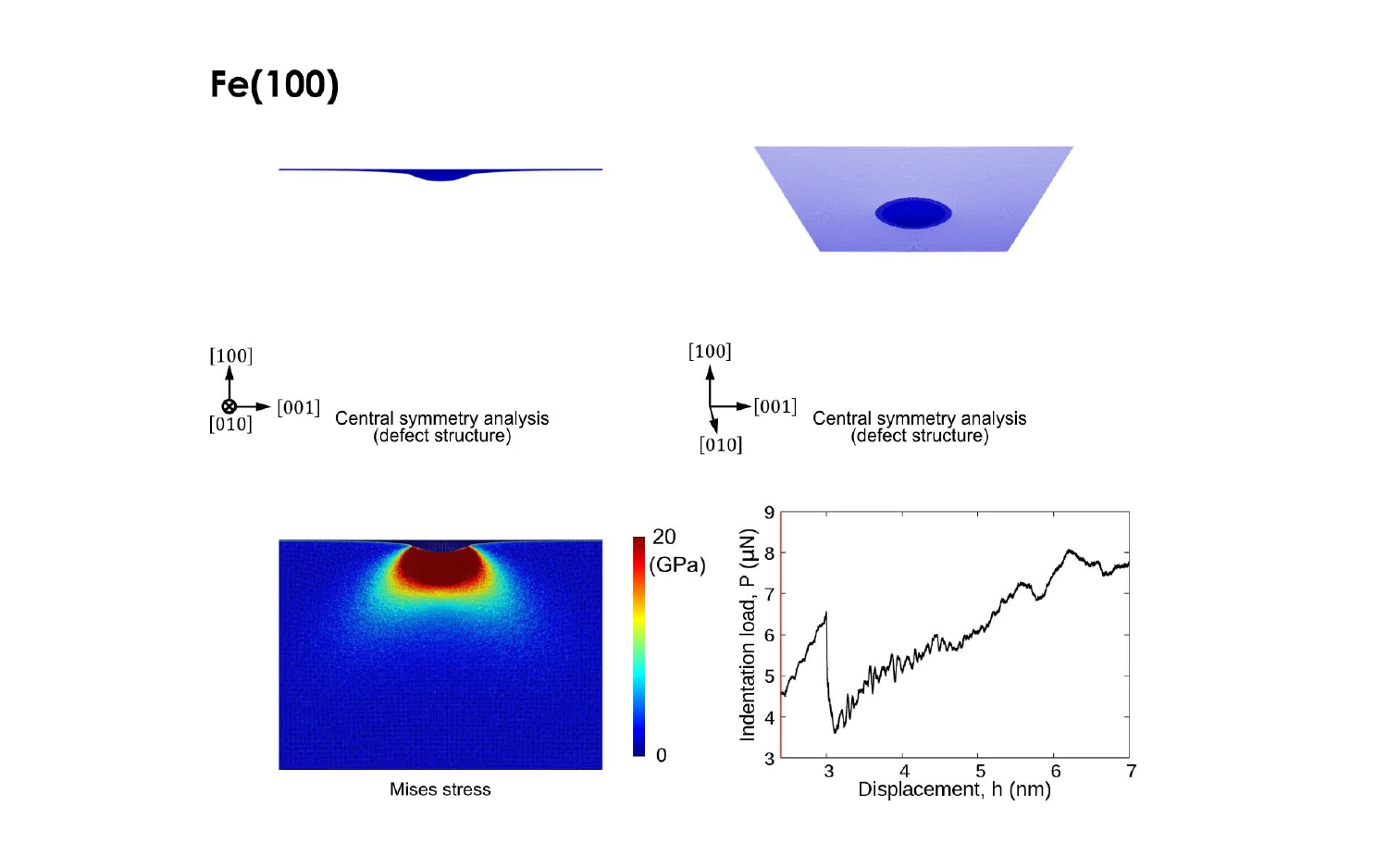

## Slide 2
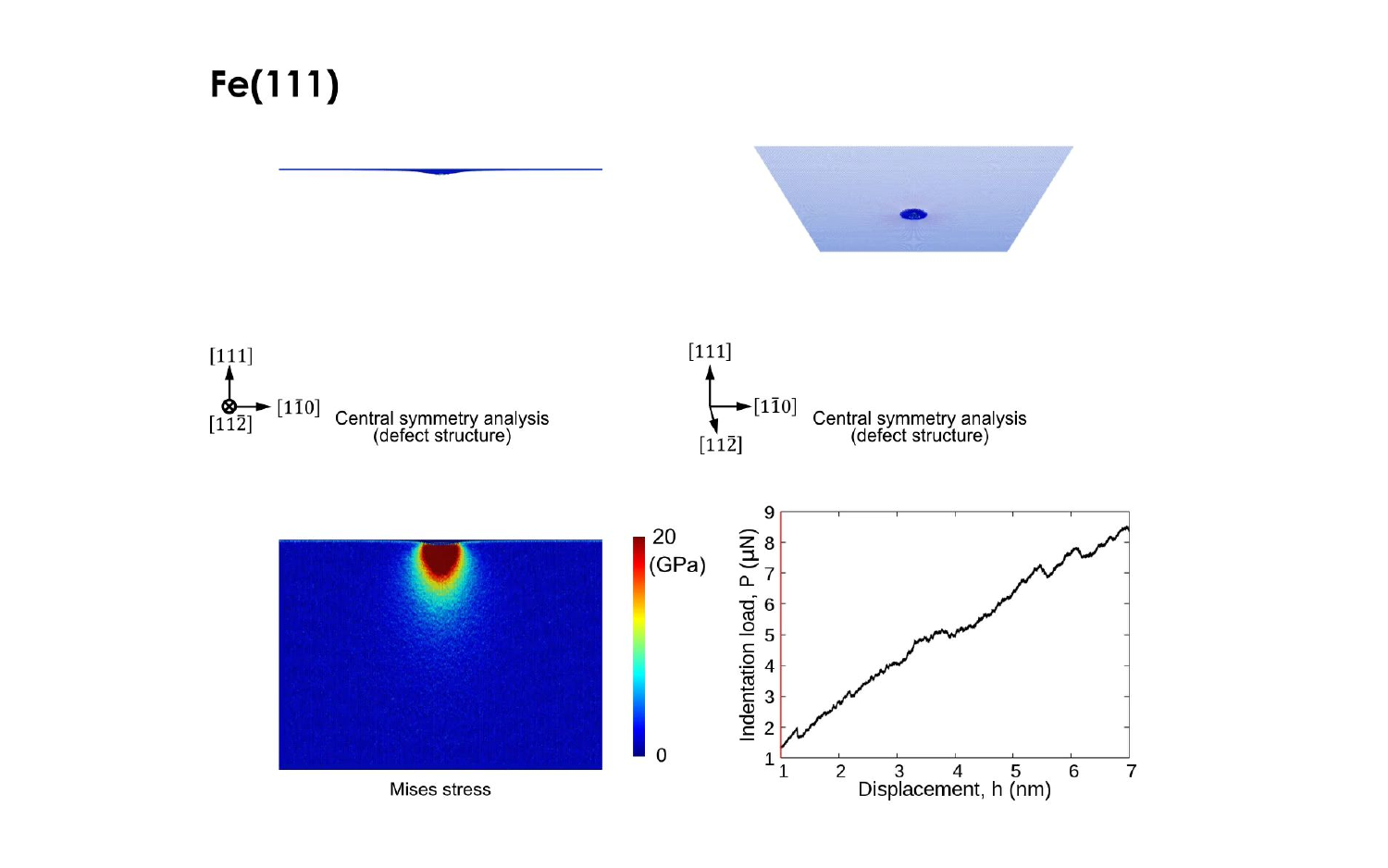

## Slide 3
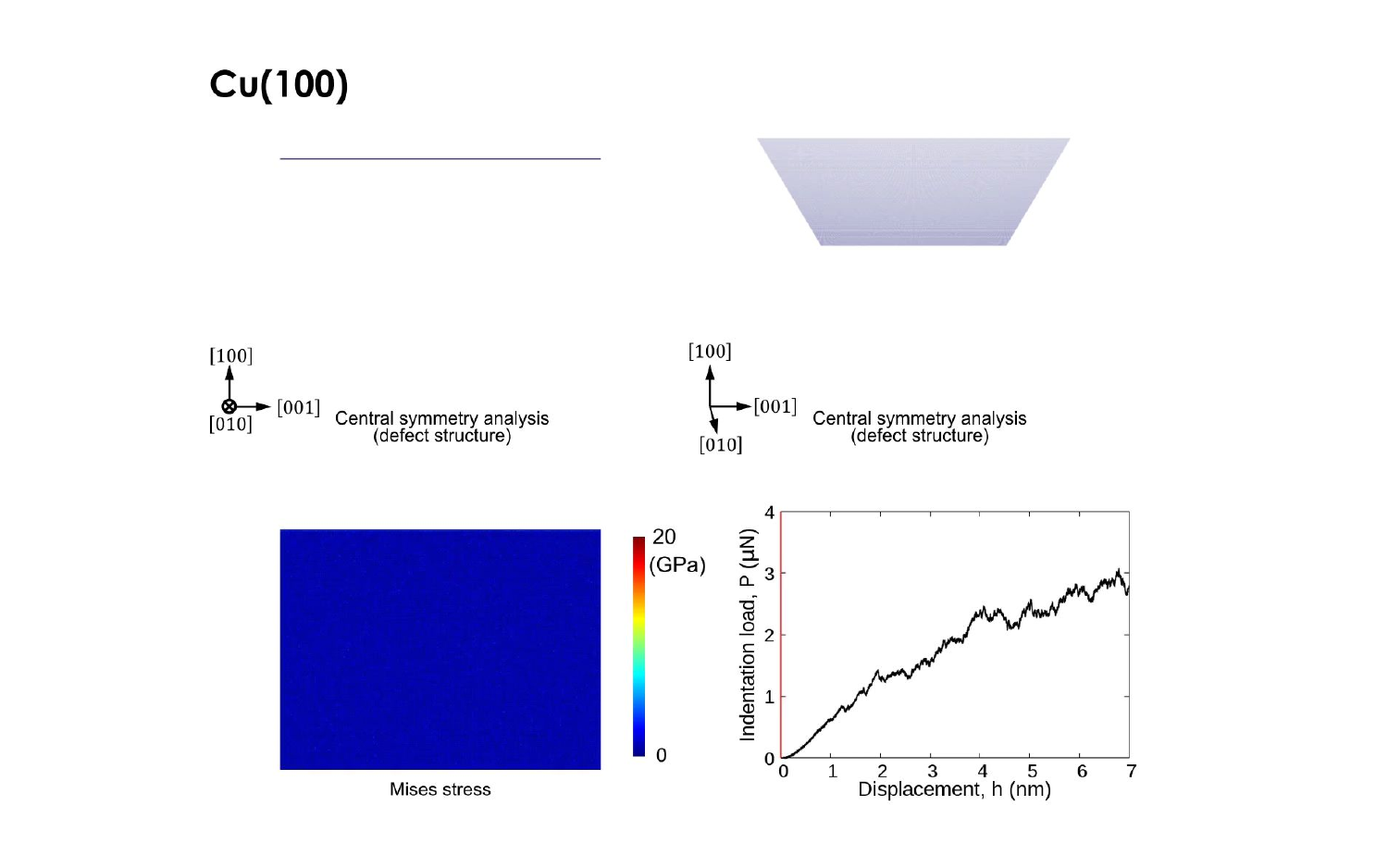

Supplement: Supplementary file 4 — Supplementary Movie 1 [file 41467_2020_17918_MOESM4_ESM.pptx]
